# Highly Efficient Luminescent Polycarboxylate Lanthanide Complexes Incorporated into Di-Ureasils by an In-Situ Sol—Gel Process

**DOI:** 10.3390/polym10040434

**Published:** 2018-04-13

**Authors:** Ming Fang, Lianshe Fu, Sandra F. H. Correia, Rute A. S. Ferreira, Luís D. Carlos

**Affiliations:** Department of Physics, CICECO-Aveiro Institute of Materials, University of Aveiro, 3810-193 Aveiro, Portugal; mingfang@ua.pt (M.F.); sandracorreia@ua.pt (S.F.H.C.); rferreira@ua.pt (R.A.S.F.)

**Keywords:** di-ureasil organic–inorganic hybrids, in-situ sol–gel synthesis, red emitters, white light emission

## Abstract

In order to prepare efficient luminescent organic–inorganic hybrid materials embedded with a lanthanide (Ln^3+^) complex with polycarboxylate ligands, Ln^3+^-doped di-ureasils with 4,4-oxybis(benzoic acid) and 1,10-phenanthroline ligands were synthesized via an in-situ sol–gel route. The resulting hybrids were structurally, thermally, and optically characterized. The energy levels of the ligands and the host-to-ion and ligand-to-ion energy transfer mechanisms were investigated (including DFT/TD–DFT calculations). The results show that these Ln^3+^-based di-ureasil hybrids exhibit promising luminescent features, e.g., Eu^3+^-based materials are bright red emitters displaying quantum yields up to 0.50 ± 0.05. The luminescent color can be fine-tuned either by selection of adequate Ln^3+^ ions or by variation of the excitation wavelength. Accordingly, white light emission with CIE coordinates of (0.33, 0.35) under 310 nm irradiation was obtained.

## 1. Introduction

Trivalent lanthanide ions (Ln^3+^) have attracted considerable attention due to their *f-f* characteristic emissions, with high emission quantum yields, narrow bandwidths, long-lived lifetimes, large ligand-induced Stokes shifts, and ligand-dependent luminescence sensitization [1]. Depending on the employed Ln^3+^ ion, the luminescence can cover a spectral range from the near-ultraviolet (UV) to the visible and even the near-infrared (NIR) regions [2]. The radiative transitions of Ln^3+^ are parity-forbidden, resulting in general in a weak absorbance and, accordingly, weak emission [3].

When suitable organic ligands coordinate the Ln^3+^ ions, however, the brightness and the luminescence quantum yield of the resultant complexes can be significantly improved upon UV light irradiation, due to Ln^3+^ luminescence sensitization or the so-called ‘‘antenna effect’’ (the ligands protect the metal ions from vibrational non-radiative quenching, inducing an effective intramolecular energy transfer) [4]. The commonly used organic ligands in Ln^3+^ complexation include β-diketones, aromatic carboxylic acids, macrocyclic ligands, and heterocyclic ancillary ligands. Despite the great interest in these complexes for a wide range of photonic applications, e.g., emitting components in multilayer organic light-emitting diodes, light concentrators for photovoltaic devices and non-linear optics [5,6,7,8], the number of effective devices is low, mainly due to their poor thermal- and photo-stabilities and low mechanical strength [9]. Therefore, in order to circumvent these drawbacks, one feasible approach is to introduce the complexes into a stable rigid matrix, e.g., a polymer or an organic–inorganic host. In fact, up to now, Ln^3+^ complexes have been incorporated into sol–gel derived matrices, such as silica- or titania-based materials [10,11], zeolites [12], mesoporous or layered materials [13,14], soft gels [15], polymers [16,17], and organic–inorganic hybrids [18,19]. The incorporation of the complexes into these matrices increased their luminescent efficiency (avoided self-quenching, due to the concentration effect), improving concomitantly their thermal- and photo-stabilities, as well as their mechanical strength and processability [20]. In this regard, emphasis must be placed on amine-functionalized di-ureasil hybrids consisting of a siliceous skeleton to which oligopolyether chains of different lengths are covalently grafted by means of urea [NHC(=O)NH] cross-links (Scheme S1, Supplementary Materials) [21,22]. As multiwavelength phosphor hosts, di-ureasils have been applied in many fields, such as white-light emitters [23,24] and luminescent solar concentrators (LSCs) [25,26]. Meanwhile, Ln^3+^-based complexes with β-diketone ligands have also been encapsulated into these di-ureasil hybrid matrices and the hybrid materials have exhibited improved stabilities and high luminescence quantum yields [27,28,29,30,31,32].

The sol–gel method is a versatile approach for synthesis of organic–inorganic hybrid materials [33,34,35,36,37,38]. Basically, there are three approaches to incorporate Ln^3+^ complexes into host materials: pre-doping, post-doping, and in-situ synthesis. While, in the pre-doping method, the preformed Ln^3+^ complex is dissolved in a solvent that is compatible with the sol in which the precursor will be hydrolyzed, in the post-doping method, the hosts (generally microporous or mesoporous materials) are soaked in a solution that contains the complex. Examples of Ln^3+^-doped organic–inorganic hybrids synthesized by the pre-doping process are reviewed in [18,19]. Moreover, post-doping examples include periodic mesoporous organosilica materials obtained by impregnation with Ln^3+^ complex solutions [39]. On the other hand, examples of hybrid materials in which the Ln^3+^ complex was formed in-situ are scarce. Examples involve heterocyclic or monodentate aromatic carboxylic ligands [40,41]. The main weakness of the pre-doping and post-doping methods is that the complexes cannot be fully homogeneously dispersed into the matrices to obtain a transparent monolithic composite, due to their low solubility and/or chemical stabilities in the sol–gel precursor solutions. Therefore, the in-situ synthesis can be not only used to mix all the compositions at a molecular level, allowing complex formation during the sol–gel process, but also applied to synthesize hybrid materials comprising Ln^3+^ coordination polymers (CPs) with multidentate ligands that are not dissolved in common organic solvents. Although some works have been reported on the grafting of Ln^3+^ complexes on sol–gel-derived hosts, the organic ligands are usually limited to monodentate carboxylate ligands and heterocyclic ligands.

Compared to Ln^3+^ complexes derived from β-diketone ligands, examples obtained from polycarboxylate ligands with multidentate coordination sites show improved thermal stability, due to their chained or network structures [42]. In these complexes, the multidentate aromatic carboxylic ligands can act not only as sensitizing agents for the Ln^3+^ ions but also as connecting linkers for the network. These polycarboxylate ligands are normally applied to prepare CPs using hydrothermal/solvothermal reactions at high temperature and high pressure conditions. An illustrative example is the 4,4′-oxybisbenzoic acid (Oba) flexible V-shaped dicarboxylate ligand (molecular structure shown in Scheme S1, Supplementary Materials) previously employed to synthesize, by hydrothermal synthesis, Ln^3+^-based CPs, [Ln(Oba)Phen(ox)_0.5_] (Ln = Eu, Gd, and Tb, Phen = 1,10-phenanthroline, ox = oxalate), Eu(Oba)_2_(Phen), and [Er/Yb(Oba)(HOba)(Phen)]*_n_*, with interesting luminescence features [43,44,45]. Since the Ln^3+^-based CPs are usually not dissolved in common organic solvents in an ambient environment, there are no reports concerning Ln^3+^ polycarboxylate complexes encapsulated into organic–inorganic hybrid matrices.

In this work, we developed an in-situ sol–gel synthesis to prepare di-ureasil hybrids doped with Ln^3+^ polycarboxylate complexes based on Oba and Phen ligands that were homogeneously distributed within the matrix. The resulting hybrid materials were structurally and optically characterized and theoretically studied by density functional theory (DFT). Whereas Eu^3+^-based materials are bright red emitters displaying high quantum yields (up to 0.50 ± 0.05), the emission color of co-doped Gd^3+^/Eu^3+^/Tb^3+^-di-ureasils can be fine-tuned by changing the excitation wavelength; consequently, white light emission was obtained.

## 2. Materials and Methods

### 2.1. Materials and Synthesis

The diamine α,ω-diaminepoly(oxyethylene-co-oxypropylene) (ED-600, commercially named Jeffamine^®^ ED-600, Huntsman, Barcelona, Spain), 3-isocyanateproplytriethoxysilane (ICPTES, 95%, Aldrich, Algés, Portugal), Oba (98%, ABCR), Phen (99%, Alfa Aesar, Karlsruhe, Germany), Eu(NO_3_)_3_·6H_2_O (Eu(NO_3_)_3_, 99.9%, Aldrich, Algés, Portugal), Gd(NO_3_)_3_·6H_2_O (Gd(NO_3_)_3_, 99.9%, Aldrich, Algés, Portugal) and Tb(NO_3_)_3_·5H_2_O (Tb(NO_3_)_3_, 99.9%, Aldrich, Algés, Portugal), and hydrochloride (HCl, Aldrich) are all commercially available. Tetrahydrofuran (THF, 99%, Sigma–Aldrich, Algés, Portugal), absolute ethanol (EtOH, Sigma–Aldrich, Algés, Portugal), and dimethylformamide (DMF, 99.99%, Fisher Chemical, Oeiras Portugal) were used as solvents. All chemicals were used as received without purifications. High-purity distilled water was used throughout experiments.

**Scheme 1:**
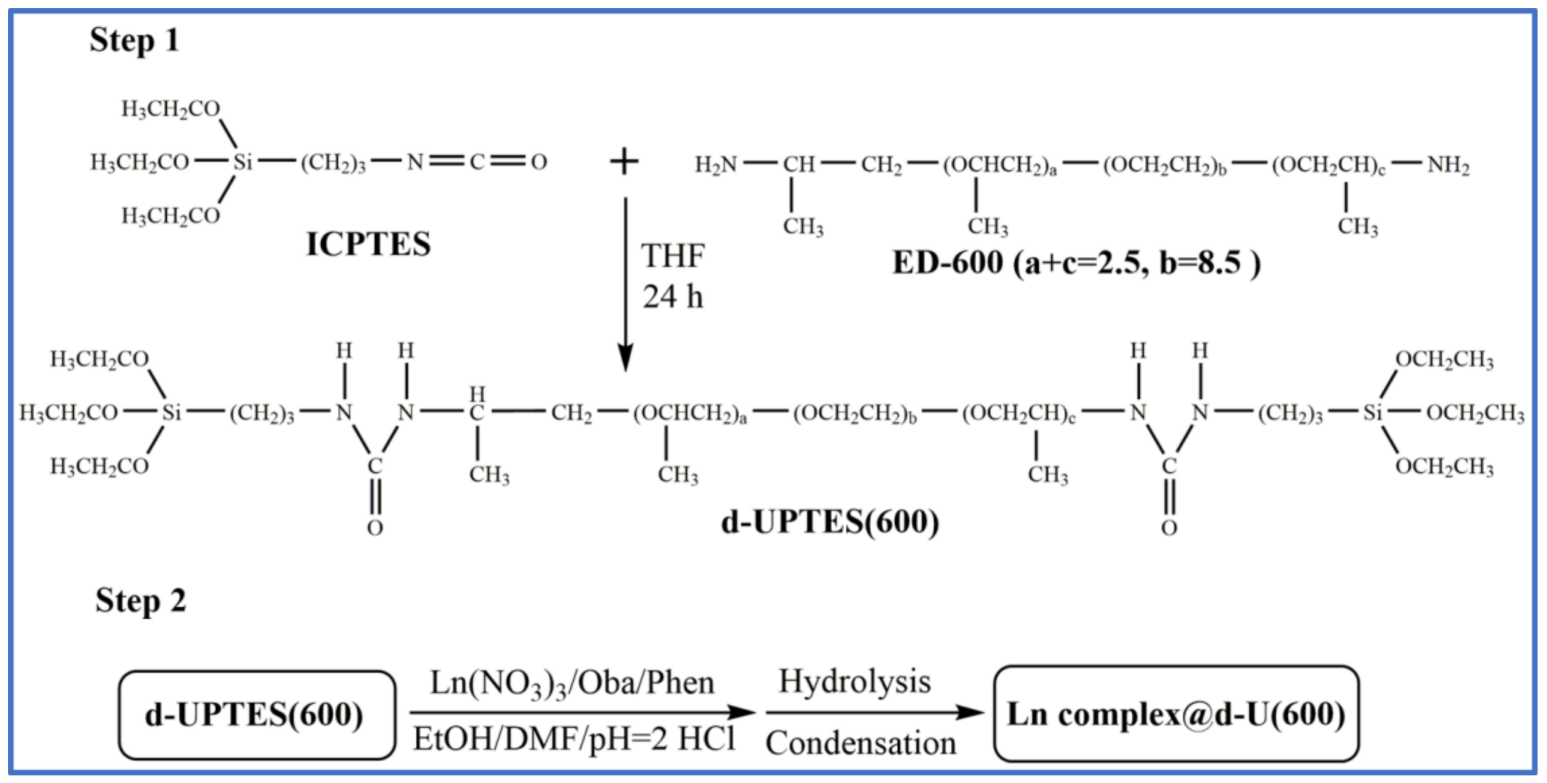
Preparation of d-U(600) containing in-situ synthesized Ln^3+^-based complexes.

The synthesis of Ln^3+^-doped di-ureasils involves two steps (Scheme 1):

(1) Synthesis of di-ureapropyltriethoxysilane (d-UPTES(600)) precursor based on the reaction between the two-side terminal amine groups of ED-600 and the isocyanate group of ICPTES; and (2) the hydrolysis and polycondensation of the precursor in the presence of HCl as catalyst and EtOH as solvent. For the synthesis of the Ln^3+^-doped di-ureasils, Ln(NO_3_)_3_ (Ln = Eu, Tb and Gd), Oba, and Phen were also added in this second step.

Step 1. Synthesis of the d-UPTES(600) precursor

The first step for preparing the d-UPTES(600) precursor has been reported elsewhere [21]. Briefly, ICPTES is added dropwise into a homogeneous mixture of ED-600 and THF under stirring at room temperature with a molar ratio of ED-600:ICPTES = 1:2. After 24 hours of stirring (at room temperature), the THF in the mixture is evaporated under vacuum and the resulting d-UPTES(600) precursor is then ready to be used in the next step.

Step 2. Synthesis of undoped and Ln^3+^-doped di-ureasils

In the second step, EtOH is used as solvent for Ln(NO_3_)_3_ (Ln = Eu, Tb and Gd) and Phen, as well as co-solvent for the d-UPTES(600) precursor (together with water). DMF is used as solvent for Oba. The undoped di-ureasil (d-U(600)) is synthesized by hydrolysis and polycondensation of d-UPTES(600) using HCl as catalyst. The Eu^3+^-doped d-U(600) is prepared similarly but excludes the incorporation of Eu(NO_3_)_3_. For synthesis of the Ln^3+^-complex-based d-U(600), typically, certain amounts of Ln(NO_3_)_3_, Oba, and Phen are mixed in a flask with EtOH and DMF solvent through ultrasonic treatment. After this procedure, the resulting mixture is transferred into the d-UPTES(600) precursor accompanied by 98.7 μL (5.5 mmol) of HCl acidized water (pH = 2.0). The molar ratio of d-UPTES(600):H_2_O = 1:6. The resulting sol is stirred for 2 h under ambient temperature before transferred into a Teflon mold. The sol is put into an oven with 60 °C until gelation occurs. During the hydrolysis process, Si–OC_2_H_5_ groups are gradually hydrolyzed to Si–OH groups and are accompanied by a condensation reaction to form cross-linking siloxane networks. The heated environment aids in the evaporation of water, EtOH, and DMF solvent. Meanwhile, Ln^3+^ complex is in-situ formed during this process.

A series of undoped and Ln^3+^-doped d-U(600) is synthesized using different dopant components and amounts, as summarized in Table S1 (Supplementary Materials). All samples are prepared based on 1.0 g (0.91 mmol) of d-UPTES(600). The resulting Eu^3+^- or ligand-doped d-U(600) is designated as *x*Eu@dU6 and xL@dU6 (L = Oba and Phen), respectively, whereas the resulting Ln^3+^ complex-doped d-U(600) is denoted as *x*Ln(Oba)*_y_*(Phen)*_z_*@dU6 (Ln = Eu, Gd, and Tb), where *x* stands for the nominal percentage of the molar ratio of the dopant to the d-UPTES(600) precursor, and *y* and *z* stand for the molar ratios of Oba and Phen to Ln^3+^ ions, respectively.

### 2.2. Characterization

Fourier transform infrared (FT-IR) spectra were obtained using a MATTSON 7000 FT-IR spectrometer to scan the sample absorbance intensity from 4000–400 cm^−1^ with 64 scans and a 4 cm^−1^ resolution. Before measurements, the prepared samples were kept in an oven at 90 °C for more than 24 h.

UV−visible (UV−Vis) absorption spectrum for Oba in DMF was measured using a dual-beam spectrometer, Lambda 950 (PerkinElmer), over a scan range of 250−400 nm and a resolution of 1 nm.

The excitation and emission spectra were recorded using a Fluorolog3^®^ Horiba Scientific (Model FL3-2T) spectroscope, with a modular double grating excitation spectrometer (fitted with a 1200 grooves/mm grating blazed at 330 nm) and a TRIAX 320 single emission monochromator (fitted with a 1200 grooves/mm grating blazed at 500 nm, reciprocal linear density of 2.6 nm mm^−1^), coupled with an R928 Hamamatsu photomultiplier, using the front face acquisition mode. The excitation source was a 450 W Xe arc lamp. The emission spectra were corrected for detection and optical spectral response of the spectrofluorimeter, and the excitation spectra were corrected for the spectral distribution of the lamp intensity using a photodiode reference detector. Low-temperature emission measurements (at 12 K) were performed using a helium-closed cycle cryostat with vacuum system measuring ca. 5 × 10^−6^ mbar and a temperature controller (Lakeshore 331) with a resistance heater. The emission decay curves were measured with the setup described for the luminescence spectra using a pulsed Xe−Hg lamp (6.0 μs pulse at half width and 20.0−30.0 μs tail).

The absolute quantum yields (*q*) were measured at room temperature using a quantum yield measurement system C9920−02 from Hamamatsu with a 150 W xenon lamp coupled with a monochromator for wavelength discrimination, an integrating sphere as a sample chamber and a multichannel analyzer for signal detection. Three measurements were made for each sample so that the average value is reported. The method is accurate to within 10%.

The photostability tests were carried out by successive UV light irradiation with a certain wavelength (450 W Xe arc lamp, entrance slit/front exit/side exit: 0.15/0.15/0.15 mm for modular double grating excitation spectrometer, entrance slit/exit slit: 0.3/0.3 mm for TRIAX 320 single emission monochromator).

The white-light emission was further quantified by the calculation of the (*x,y*) Commission Internationale de l’Eclairage (CIE) emission color coordinates for the 2° observer. The color correlated temperature (CCT), the temperature of a blackbody radiator emitting the same color of the light source, typically ranges from 2500 K to 6500 K for lighting applications. The CCT values can be calculated through the following polynomial function [46]:

(1) $$CCT=at^{3}+bt^{2}+ct+d$$

where *a* = 449, *b* = 3525, *c* = 6823.3, *d* = 5520.33 and *t* is given by the following equation:

(2) $$t=\frac{\left(x-x_{e}\right)}{\left(y_{e}-y\right)}$$

with (*x_e_*,*y_e_*) = (0.3320,0.1858).

### 2.3. Theoretical and Computational Details

The geometrical structures of the ground state (S_0_) and the lowest-lying triplet state (T_1_) of Oba were optimized by restricted and unrestricted density functional theory (DFT), respectively, using B3LYP/6-311G(d,p) level of theory [47,48], and the energies were calculated at the B3LYP/def2-TZVP level of theory. Harmonic vibrational frequencies were calculated at the same level of theory on the optimized structures to ascertain that each configuration was a minimum on the potential energy surface. Based on the optimized ground state equilibrium geometries, the vertical transitions with five low-lying singlet states and the first excited state were calculated by a time-dependent DFT (TD-DFT) approach. The solvent effect was also considered with the polarized continuum model (PCM) in DMF medium throughout the calculations. Visualization of the molecular orbital (MO) was achieved with Multiwfn combined with VMD program [49,50]. Contributions of different atoms to the frontier molecular orbital (FMO) were obtained by Multiwfn using Mulliken population analysis. All calculations were performed without any symmetry restrictions using Gaussian 09 [51].

## 3. Results and Discussion

### 3.1. Powder XRD Patterns

The XRD pattern of the 6Eu(Oba)_1.5_(Phen)_1.5_@dU6 was compared with that of the undoped d-U(600) (Figure S1, Supplementary Materials). Both exhibited a broad band centered around 21° associated with the presence of amorphous siliceous domains, whose second diffraction order appeared as an even broader weak hump, also both around 39° to 44° [52,53]. Additionally, the typical shoulder from 9° to 15° was clearly discernable, arising from other intra-siloxane domains’ in-plane ordering, with a characteristic distance of ca. 7.8 ± 0.5 Å, independent of the existence of dopant. According to the Bragg law [54], *d* = λ/2sinθ, where λ is the wavelength of the incident radiation, structural unit distances of *d* = 4.2 ± 0.1 Å were estimated for both hybrids, similarly to previously reported works [55,56]. Furthermore, the XRD patterns of hybrids doped with the amount in the range of 2–10 mol % mixture demonstrated an unaltered host local structure (Figure S1, Supplementary Materials).

### 3.2. FT-IR Spectroscopy

**Figure 1:**
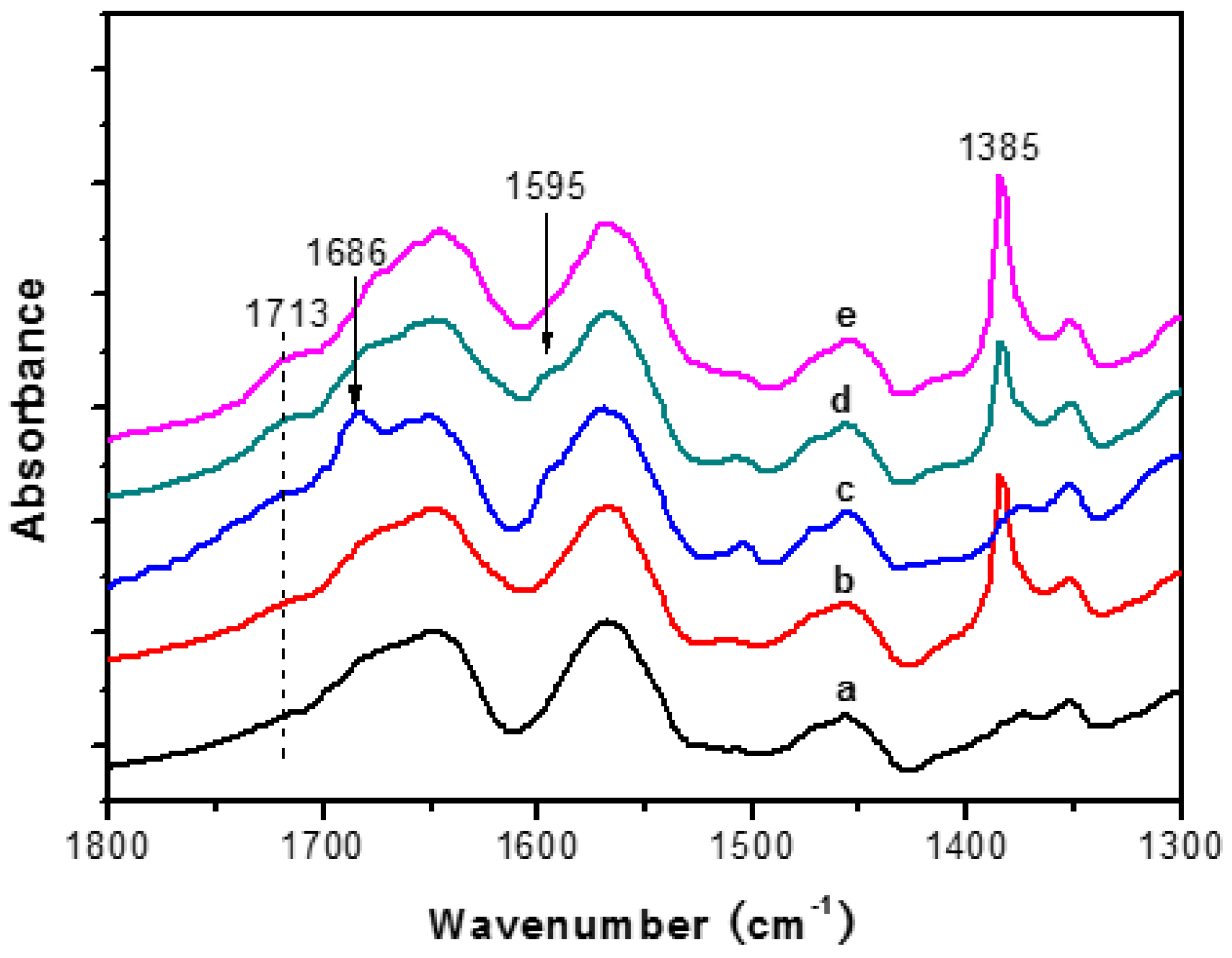
FT-IR spectra of (**a**) d-U(600); (**b**) 6Eu@dU; (**c**) 6Oba@dU6; (**d**) 6Eu(Oba)_1.5_@dU6; and (**e**) 6Eu(Oba)_1.5_(Phen)_1.5_@dU6.

Figure 1 shows the FT-IR spectra of undoped d-U(600) and its analogues doped with Eu(NO_3_)_3_ and organic ligands. As is well known, the amide I envelope (1800–1600 cm^−1^) reflects the presence of hydrogen-bonded structures involving the interaction between the N–H moieties of the urea linkages and the carbonyl oxygen atom of a neighbor urea group or the ether oxygen atoms of the polymer segments, whereas the amide II mode (1600–1500 cm^−1^) is a mixed contribution of the N–H in-plane bending, C–N stretching, and C–C stretching vibrations [21]. The amide I and amide II bands of d-U(600) were deconvoluted, and three components were isolated at 1713, 1675, and 1642 cm^−1^ for the former and one peak at 1569 cm^−1^ for the latter (Figure S2a, Supplementary Materials). While the components at 1713 and 1675 cm^−1^ are ascribed to the vibrations of urea-polyether hydrogen-bonded structures, the component at 1642 cm^−1^ is due to the formation of the urea–urea hydrogen-bonded associations [57], which suggests that neither C=O from urea moieties nor N–H groups are left free in the hybrid.

When 6 mol% Eu(NO_3_)_3_ was incorporated into d-U(600) (here the ratio of the (OCH_2_CH_2_) units in d-U(600) per Eu^3+^ ion (*n’*) is 142), the profiles of amide I and amide II remained unchanged, implying that the urea linkages are not involved in the coordination of the Eu^3+^ ions, a similar situation when Eu(ClO_4_)_3_ with *n’* = 232 was introduced into d-U(600) [57]. On the other hand, compared to the spectrum of undoped d-U(600) (Figure 1, Curve a), a distinct peak appeared at 1385 cm^−1^ (Figure 1, Curve b), the characteristic N–O stretching vibration band of NO_3_^−^ anion, as shown in Figure S2 (Supplementary Materials). Therefore, the ether oxygen atoms of the polymer chains in d-U(600) are responsible for the presence of the “spectroscopically free” NO_3_^−^ moieties.

The vibration bands of asymmetry –COOH and of symmetry –COO^−^ groups that appeared at 1686 and 1595 cm^−1^, respectively, can be clearly discerned [45,58] on the spectrum of 6Oba@dU6. When d-U(600) codoped with Eu(NO_3_)_3_ and organic ligands, the decrease in intensity in, or even the disappearance of, these two bands indicates that Eu^3+^ ions coordinate with the organic ligands. For comparison of the relative intensity, the amide II bands were normalized for all spectra in Figure 1. The band intensity of NO_3_^−^ anions (1385 cm^−1^) of 6Eu(Oba)_1.5_(Phen)_1.5_@dU6 is larger than that of 6Eu(Oba)_1.5_@dU6, which can be explained by the fact that the Eu^3+^ ions not only form bonds with Oba but also coordinate with Phen, leaving free NO_3_^−^ moieties. These evidences imply that Eu^3+^ ions prefer to form bonds with Oba and Phen compared with the NO_3_^−^ groups. Furthermore, the intensities of the peak at 1713 cm^−1^ increase when Eu(NO_3_)_3_ and the organic ligands are codoped into d-U(600) relative to only the Eu(NO_3_)_3_-doped analogue, indicating that Eu^3+^ ions tend to coordinate with organic ligands and more urea–polyether hydrogen-bonded associations [57].

### 3.3. ^29^Si MAS and ^13^C CP NMR Spectra

The ^29^Si MAS NMR spectrum of 6Eu(Oba)_1.5_(Phen)_1.5_@dU6 is displayed in Figure S3a (Supplementary Materials). The broad band consists of T^1^ (at ~–50.0 ppm), T^2^ (at ~–59.0 ppm), and T^3^ (at ~–66.0 ppm) characteristic units [59,60]. The conventional T^n^ (*n* = 1, 2, and 3) represents the reaction levels of Si surrounding and *n* is the number of Si-bridging oxygen atoms. (SiO)_2_Si(CH_2_)_3_OH and (SiO)_3_Si(CH_2_)_3_ are two typical types of T^2^ and T^3^, respectively. The negligible of T^1^ signals reflects the high degree of condensation. The signals between ca. −90 and −130 ppm are assigned to (≡SiO)_2_Si(OH)_2_ (Q^2^, geminal silanols), (≡SiO)_3_SiOH (Q^3^, single silanol), and (≡SiO)_4_Si (Q^4^, siloxane) local environments indicating the pre-hydrolysis of the d-UPTES(600) precursor [61].

According to ^13^C CP MAS NMR spectrum of the above-doped hybrid in Figure S3b (Supplementary Materials), the strong signal at 70.5 ppm is assigned to hybrids –(OCH_2_CH_2_)– group, whereas the shoulder at 75.0 ppm is attributed to the –OCH group. The relatively weak resonances at 17.4 and 159.0 ppm connect tightly with different –CH_3_ groups and urea C=O groups, respectively [56,62]. The feature signals of ICPTES, usually appeared at 11.2, 24.8, and 43.6 ppm [63], are not detected in these spectra, which implies a complete reaction of the precursor. The results are consistent with ^29^Si MAS NMR spectra results, as well as a previous publication [56].

### 3.4. TG and Thermal Stability Analyses

The thermal stabilities of d-U(600) and 6Eu(Oba)_1.5_(Phen)_1.5_@dU6 were examined in the temperature range from 75 to 800 °C. The weight losses show a similar decomposition process (Figure S4, Supplementary Materials). Before 165 °C, the unimportant weight loss is mainly ascribed to the evaporation of a small amount of absorbed water and/or ethanol/THF/DMF solvents, whereas, after 165 °C, the Ln^3+^-doped hybrids start to decompose with a rapid weight loss from 200 to 400 °C. In addition, the starting degradation temperature of the undoped d-U(600) is at 175 °C [31], which indicates that the thermal stabilities of hybrids can be slightly influenced by their interactions with the Eu^3+^ complexes. From around 200 to 600 °C, the weight losses are mainly due to the decomposition of the polymer chains of the di-ureasil host and the Eu^3+^ complexes, and SiO_2_ and Eu_2_O_3_ are finally formed. The final weights are 14.4% and 15.8% of the initial ones for d-U(600) and 6Eu(Oba)_1.5_(Phen)_1.5_@dU6, respectively, which are consistent with the theoretical values of 13.5% of d-U(600) with complete hydrolysis/condensation reaction and without any solvents.

### 3.5. Electronic Properties

DFT/TD-DFT calculations were performed for the Oba ligand aiming at estimating the energy of the first excited singlet (S_1_) and triplet (T_1_) states. To provide the framework for the excited states TD-DFT results, it will be useful to examine the electronic structure in terms of the highest occupied molecular orbital (HOMO) and the lowest unoccupied molecular orbital (LUMO) (Figure S5, Supplementary Materials). The contributions from individual atoms to HOMO and LUMO, as well as their energies, are presented in Figure S6 and Table S2 (Supplementary Materials). Population analyses reveal that the HOMO is mainly localized on the ether oxygen atom and phenyl rings, whereas the LUMO is predominantly spread on the two phenyl rings and carbonyl groups. The first vertical transition with the strongest intensity dominantly (98.5%) involves HOMO→LUMO. Therefore, the absorption can be attributed to the transitions from the lone pair electron of ether oxygen atom to phenyl rings (*n*→π*) and from phenyl rings to carbonyl groups (π→π*). This intramolecular charge transfer can be regarded as local excitation in nature. The Oba-related excited state energy levels for S_1_ and T_1_ were calculated to be around 31,950 cm^−1^ (313 nm) and 27,700 cm^−1^ (361 nm), respectively. The S_1_ energy compares well with experimental UV–Vis absorption spectrum of the Oba ligand in DMF solution (Figure S7, Supplementary Materials), whose absorption edge is around 33,300 cm^−1^ (300 nm). In what concerns T_1_, experimental validation can be found through the measurement of the phosphorescence spectrum of the 6Gd(Oba)_1.5_@dU6 at 12 K. This procedure is valid because the energy of the Gd^3+^ excited levels is much higher than that of Oba triplet states, inhibiting any ligand-to-metal energy transfer process and enabling T_1_ phosphorescence with a maximum peak position at 26,250 cm^−1^ (381 nm) (Figure S8, Supplementary Materials).

### 3.6. Photoluminescence

**Figure 2:**
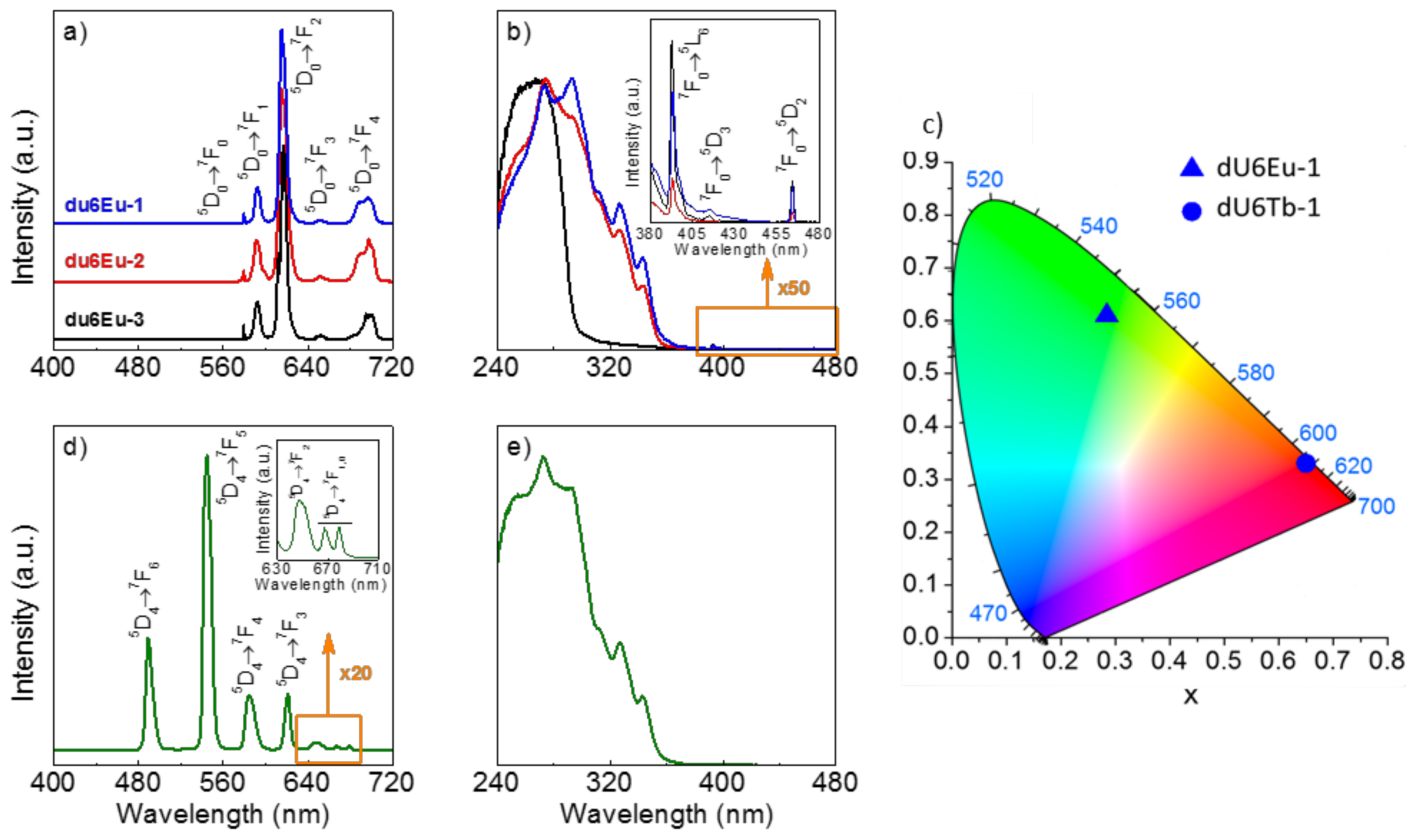
Room temperature (**a**) emission spectra excited at 275 nm (**dU6Eu-3**) and 295 nm (**dU6Eu-1**, **dU6Eu-2**); (**b**) excitation spectra monitored at 615 nm (**dU6Eu-1** and **dU6Eu-2**) and 617 nm (**dU6Eu-3**); (**c**) CIE (1931) (*x*,*y*) chromaticity diagram showing the color coordinates of **dU6Tb-1** and **dU6Eu-1**; and (**d**) emission; and (**e**) excitation spectra of **dU6Tb-1** excited at 295 nm and monitored at 545 nm, respectively.

**Figure 3:**
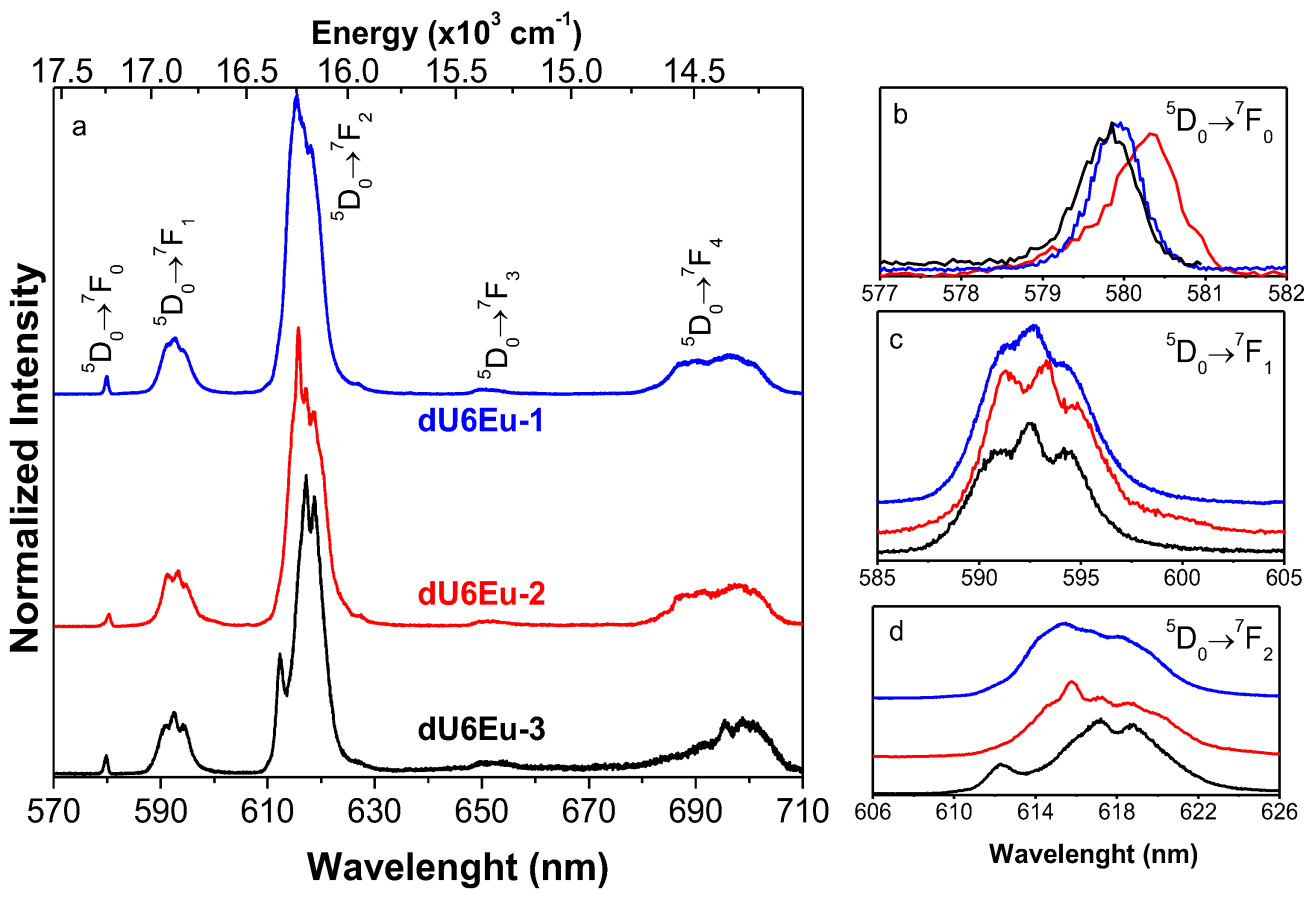
(**a**) High-resolution emission spectra (12 K) of **dU6Eu-1** (excited at 275 nm), **dU6Eu-2** and **dU6Eu-3** (excited at 295 nm) and (**b**–**d**) magnification of the corresponding ^5^D_0_→^7^F_0–2_ transitions.

Figure 2a shows the emission spectra of 6Eu(Oba)_1.5_(Phen)_1.5_@dU6 (**dU6Eu-1**) and those from the hybrids with single ligand-based complexes 6Eu(Oba)_1.5_@dU6 (**dU6Eu-2**) and 6Eu(Phen)_1.5_@dU6 (**dU6Eu-3**). All spectra are composed of a series of straight lines assigned to the Eu^3+ 5^D_0_→^7^F_0–4_ transitions, whose emission color coordinates correspond to the pure red with (*x*,*y*) CIE color coordinates of (0.65, 0.33), as illustrated in Figure 2c. The energy and full-width-at-half-maximum (fwhm) of the intra-4f^6^ transitions is independent of the excitation wavelength (Figure S9, Supplementary Materials), indicating the presence of a single Eu^3+^ average local environment within each sample. The absence of the ligands’ triplet-related emission suggests an efficient ligand-to-Eu^3+^ energy transfer. In Figure 3, the corresponding high-resolution emission spectra of Eu^3+^ ions (12 K) confirm the single low-symmetry sites of Eu^3+^ ions for each sample due to the all single non-degenerated ^5^D_0_→^7^F_0_ and Stark splitting of ^5^D_0_→^7^F_1–2_ transitions.

Further evidence of the role of the ligands in the energy transfer processes can be inferred by the measurement of the excitation spectra monitored within the Eu^3+^‘s more intense transition (^5^D_0_→^7^F_2_), shown in Figure 2b. The spectra of the **dU6Eu-1** resembles that of the **dU6Eu-2** being dominated by a broad band in the UV (240–360 nm) assigned to transitions predominantly involving the Phen singlet intraligand π→π* transitions, whose S_1_ is documented in the literature to be about 31,000 cm^−1^ (322 nm) [64]. The relative intensity variations may arise from contributions of the Oba-ligand excited S_1_ state in the low-wavelength region (240–280 nm), clearly identified as the excitation spectrum **dU6Eu-3**, whose onset at ~300 nm resembles that observed for the UV–Vis absorption spectrum of the Oba ligand in DMF (Section 3.5). Moreover, the intrinsic d-U(600) excited states overlap the Oba and Phen excited states’ spectral region (Figure S10, Supplementary Materials), so its involvement cannot be neglected. All spectra also reveal the presence of a series of intra-4f^6^ lines attributed to transitions between the ^7^F_0_ and the ^5^L_6_, ^5^D_3,2_ excited states. The very-low relative intensity of these lines compared with that of the ligand-related broad band points out a more efficient ligand-sensitization process compared with direct intra-4f^6^ excitation.

Further evidences of changes in the Eu^3+^-local environment are found in the ^5^D_0_ emission decay curves monitored around the ^5^D_0_→^7^F_2_ transition and excited at 275 or 295 nm. All curves reveal a single exponential behavior (Figures S11b–d), in good agreement with the presence of a single average Eu^3+^ local environment, but with distinct emission decay rates (Table 1). The distinct ^5^D_0_ lifetime values (τ) may be explained in terms of the radiative (*k_r_*) and nonradiative (*k_nr_*) transition probabilities and ^5^D_0_ quantum efficiency (*η*), *η* = *k_r_*/(*k_r_* + *k_nr_*), using a procedure based on the ^5^D_0_→^7^F_0–4_ integrated areas and ^5^D_0_ lifetime, recorded at room temperature for the same excitation wavelength. Using LUMPAC software [65], we observed that the larger τ value was found for the **dU6Eu-1** results from the cooperative effect of Oba and Phen ligands, which yield both an increase in the *k_r_* (5 and 10% relative to those of **dU6Eu-2** and **dU6Eu-3**, respectively) and a decrease in the *k_nr_* (21% and 31% relative to those of **dU6Eu-2** and **dU6Eu-3**, respectively) (Table 1). The number of water molecules (*n_w_*) coordinated to Eu^3+^ ions is determined using the empirical formula of Supkowski and Horrocks, *n_w_* = 1.11 × [(*k_exp_ − k_r_ −* 0.31)] (see Supplementary Material for details), where *k_exp_* is the experimental transition probability (*k_exp_* = *τ*^−1^), yielding *n_w_* values below 1 in all the cases, indicating that no water molecules can be found in the Eu^3+^-local environment.

To further quantify the role of the ligands on the emission properties, *q* was measured as a function of the excitation wavelength, namely, at 275 nm involving the Oba ligand and 275–325 nm, to selectively excite the Phen ligand (Table 1). The quantum yield values of **dU6Eu-1** are smaller than those of **dU6Eu-2**, independently of the excitation wavelength, although larger than that of **dU6Eu-3** at 275 nm. This latter aspect leads us to suggest that the changes in the Eu^3+^-local coordination shell due to the presence of the Oba and Phen ligands yield less efficient sensitization energy transfer mechanisms, despite the positive cooperative effect that yields the above-mentioned changes in the ^5^D_0_ radiative (increase) and non-radiative (decrease) paths.

The *q* values in Table 1 are larger than those known for isolated complexes involving Oba ligand, namely Eu_2_(Oba)_3_(H_2_O)_6_·3H_2_O (0.03 excited at 318 nm) and [Eu_2_(Oba)_3_(H_2_O)_5.5_ 0.5H_2_O] (0.04 excited at 318 nm) [66] or Phen ligand in Eu_x_La_1−*x*_(Phen)_2_(NO_3_)_3_ (0.39 excited at 255 nm) [67]. Nevertheless, the [Eu(Oba)Phen(ox)_0.5_]*_n_* reveals a larger *q* value (0.51) [43] when compared with that of **dU6Eu-1**.

Focusing on siloxane-based hybrid materials, we notice that the *q* values in Table 1, compared with those in the literature for tri-ureasil hosts incorporating Eu^3+^-based complexes, are smaller than those involving Eu(tta)_3_ephen (tta = 2-thenoyltrifluoracetonate; ephen = 5,6-epoxy-5,6- dihydro-[1,10]phenanthroline), 0.63 ± 0.06 [68], and Eu(tta)_3_·2(H_2_O), 0.85 ± 0.06 [26], both excited at 360 nm. Nevertheless, the quantum yield values are larger than those found for Eu(tta)_3_Phen incorporated into a siloxane-based biohybrid host including short poly-ε-caprolactone segments 0.22 ± 0.02 [69].

Focusing on the role of the Oba and Phen on the optical properties, a series of Eu^3+^-based hybrids were prepared varying their relative contribution (Table S1, Supplementary Materials). The photoluminescence features of emission, excitation spectra, and quantum yield (Figures S9 and S10 and Table S3, Supplementary Materials) are analogous to those detailed for **dU6Eu-1**. Therefore, the search for new optical properties will be performed using a Phen/Oba ratio analogous to that in **dU6Eu-1**. In particular, the replacement of Eu^3+^ by Tb^3+^ ions yields a hybrid material (**dU6Tb-1**) able to emit light in the green spectral region with CIE (*x*,*y*) color coordinates of (0.28, 0.61) due to the contribution of the Tb^3+ 5^D_4_→^7^F_6-0_ transitions (Figure 2c,d). Such intra-4f^8^ transitions arise from efficient ligands→Tb^3+^ energy transfer, as evidenced by the excitation spectrum monitored within the more intense Tb^3+ 5^D_4_→^7^F_5_ transition (Figure 2e), which is also dominated by the broad band already observed for **dU6Eu-1**, which is ascribed to the Phen and Oba excited states. The ^5^D_4_ emission decay curve of **dU6Tb-1** was monitored around 544 nm, excited at 275 nm, revealing a single exponential behavior (Figure S11e) characterized by a lifetime value of 0.97 ± 0.05 ms. The emission quantum yield of **dU6Tb-1** is stable within 0.23 to 0.21 ± 0.02 under excitation at 275–295 nm but decreases to 0.10 ± 0.01 under excitation at 325 nm. These values are smaller than those of isolated complexes [Tb(Oba)Phen(ox)_0.5_]*_n_* (0.42) [43], [Tb_2_(Oba)_3_(H_2_O)_6_·3H_2_O]*_n_* (0.72 excited at 305 nm), and [Tb_2_(Oba)_3_(H_2_O)_5.5_·0.5H_2_O]_*n*_ (0.66 excited at 305 nm) [66].

**Figure 4:**
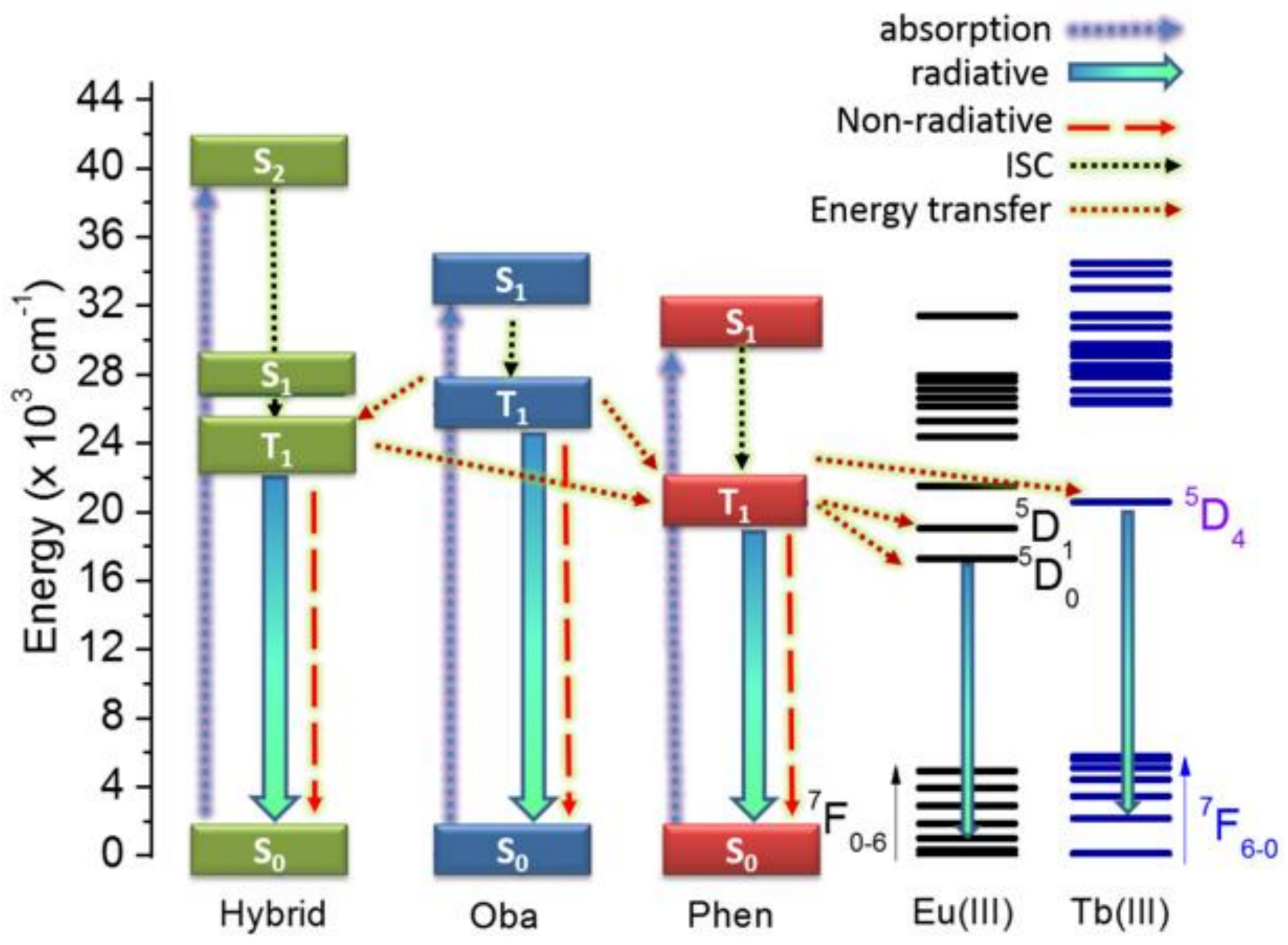
Partial energy diagram highlighting the main potential relaxation pathways in **dU6Eu-1**, **dU6Tb-1**, and **dU6GdEuTb-1** hybrids.

**Figure 5:**
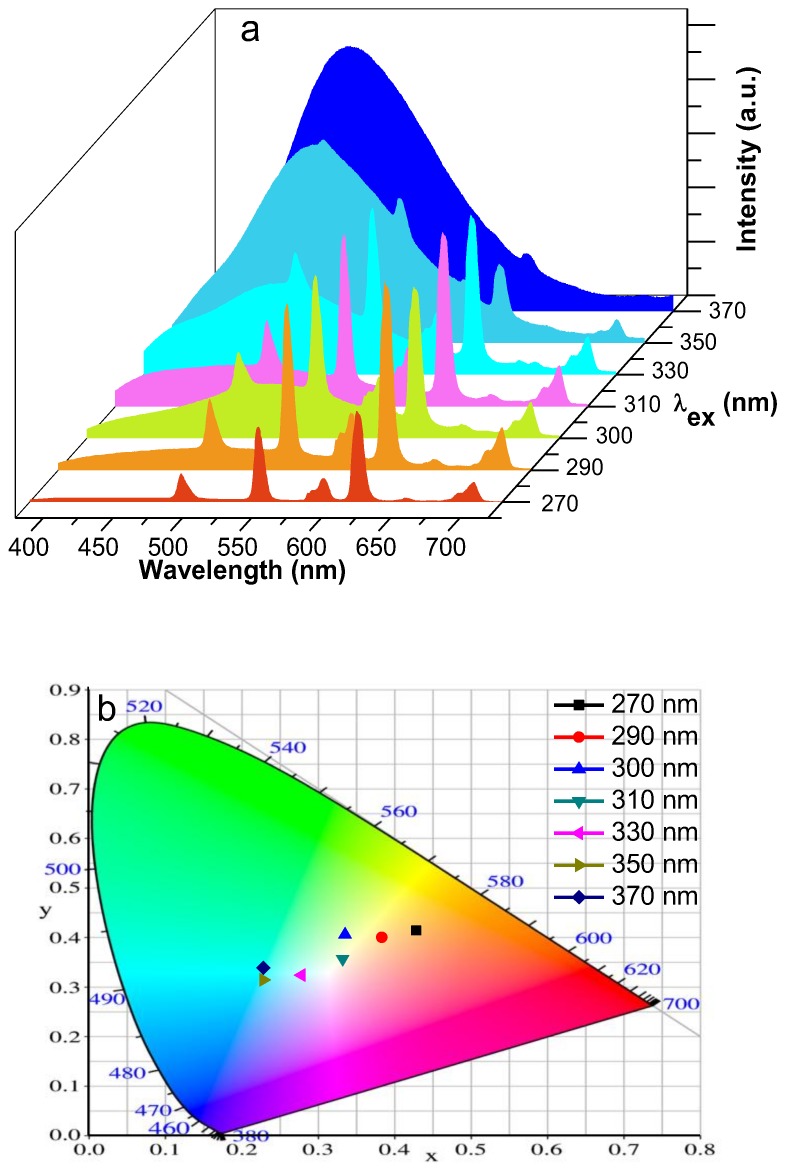
(**a**) Emission spectra and (**b**) CIE chromaticity coordinates of **dU6GdTbEu-1** excited at different wavelengths from 270 to 370 nm.

To gain further insight into the role of the Oba and Phen ligands in the energy transfer processes involving the Eu^3+^ and the Tb^3+^ ions, an energy diagram is given in Figure 4. The S_1_ and T_1_ energy values of the Oba ligand were taken from the experimental results mentioned in Section 3.5. The S_1_ energy of Phen was set to be about 31,000 cm^−1^ (322 nm) [64], and T_1_ was inferred from the emission spectrum of the 3Gd_0.91_Eu_0.05_Tb_0.04_(Oba)_1.5_(Phen)_1.5_@dU6 (**dU6GdTbEu-1)** (Figure 5) being around 488 nm (20,492 cm^−1^). The lowest energy singlet and triplet excited states of the d-U(600) host were taken from the literature [28].

The energy gap (ΔE) values between S_1_ and T_1_ are 7050 and 10,508 cm^−1^ for Oba and Phen, respectively. As ΔE is larger than 5000 cm^−1^, intersystem crossing (ISC) processes for both ligands cannot be neglected, in accordance to Reinhoudt’s empirical rule [70]. To get an efficient energy transfer from T_1_ to Ln^3+^ ions, it is well known that the triplet state energy level of the ligands should be in a suitable range. Latva et al. have investigated Eu complexes with a series of ligands (the T_1_ levels between 19,000–28,000 cm^−1^) and concluded that the triplet state energy level of 20,000–23,000 cm^−1^ for the ligands can efficiently sensitize Eu emission [71]. With respect to the T_1_ levels of Phen (20,492 cm^−1^) and Oba (26,250 cm^−1^), although both can match the ^5^D_1_ and ^5^D_0_ resonance levels of Eu^3+^ ions, the T_1_ energy level of Phen demonstrates a more efficient energy transfer path than that of Oba, which results in η = 0.43, for **dU6Eu-2** under 295 nm irradiation, and η = 0.37, for **dU6Eu-3** under 275 nm irradiation, as shown in Table S3 (Supplementary Materials). Referring to all the energy levels for the ligands, d-U(600) hybrid matrix and Eu^3+^ excited states (Figure 4), since the T_1_ energy level of Oba is higher than that of Phen by around 5700 cm^−1^, it can sensitize the Eu^3+^ emission through a two-step intermolecular energy transfer process, namely, between (i) T_1_ of Oba to T_1_ of Phen, followed by T_1_ of Phen-to-Eu^3^^+^ energy transfer, and (ii) between T_1_ of Oba and hybrid, followed by hybrid-to-T_1_ of Phen-to-Eu^3^^+^ and hybrid-to-Eu^3^^+^ energy transfer [31]. These results show the crucial role of Oba in the population of the Eu^3^^+^ emitting levels through the hybrid-related states.

As illustrated in the diagram in Figure 2c, the emission color is chemically tuned by the adequate selection of the Ln^3+^ ion, namely Tb^3+^ (green) and Eu^3+^ (red). The combination of the two ions in a single hybrid material opens the possibility to change the emission color using external physical parameters, such as the excitation wavelength. Thus, based on the previous results, a mixed Eu^3+^/Tb^3+^-doped d-U(600) hybrid with an Oba/Phen ratio analogous to that in **dU6Eu-1** was prepared. To tune the energy-transfer processes and enable the ligands related emission, this sample was also doped with Gd^3+^ ions as a strategy to enable the observation of the ligands/hybrid intrinsic emissions as the efficient energy transfer observed from those to the Eu^3+^ and Tb^3+^ ions, which will be inhibited due to the much higher excited levels of Gd^3+^ than the first triplet states of the ligands. Therefore, the ligands that are anchored by Gd^3+^ ions will emit rather than being able to transfer energy to the Gd^3+^ ions, adding the blue component to the red (Eu^3+^) and green (Tb^3+^) contributions, as shown in Figure 5. The peak position of the observed broad band around 488 nm (20,492 cm^−1^) is very close to the T_1_ state reported for the Phen ligand around 22,100 cm^−1^ (452 nm) [64]. Moreover, comparing the T_1_ energy peak position excited at 370 nm for the **dU6GdTbEu-1** (488 nm, 20,492 cm^−1^) with that of the undoped hybrid host (Figure S12, Supplementary Materials) under analogous excitation, we observed a significant blue-shift (58 nm, 2764 cm^−1^) for the d-U(600) emission spectrum. In addition, the energy of the band in Figure 5a is independent of the excitation wavelength, and the typical red shift of the emission peak position as the excitation wavelength increases, known from the di-ureasil host [31,56], reinforces this component, which arises from the T_1_ of the Phen ligand. A possible contribution from the Oba triplet state is not predictable, as the triplet state is characterized by higher energies (Figure S8, Supplementary Materials) and it is not excited by energy lower than 300 nm (Figure 2b and Figure S7, Supplementary Materials). Thus, the broad band in Figure 5a is mainly ascribed to the Phen ligands’ triplet states.

Interestingly, the cooperative effect of the emission arising from the Phen ligand and the Tb^3+^ and Eu^3+^ ions allows for the tuning of the hybrid’s relative emission intensity as the excitation wavelength increases from 270 to 370 nm. As a result, the emission color varies from the orange-reddish spectral region, with a CCT of 3200 K and color coordinates of (0.43, 0.41) at 270 nm, to a bluish-green region, (0.23, 0.34) at 370 nm, crossing the center of the diagram ascribed to the pure white light region with a CCT of 5600 K, coordinates of (0.33, 0.35), and a quantum yield of *q* = 0.03 ± 0.01 under an excitation at 310 nm. The measured quantum yield values are given in Table S4, Supplementary Materials.

The cooperative effect of Oba and Phen ligands is at around an excitation of 275 nm. Under preferential excitation into the Phen ligand (325–345 nm), the quantum yield of **dU6Eu-1** is analogous to that of **dU6Eu-2**. The fact that quantum yield is maximum at 295 nm can be explained as this excitation wavelength corresponds to the maximum contribution of the Phen ligand for the excitation spectra, still overlapping the Oba ligand, reinforcing the relevance of the cooperative role of the two ligands to boost the Eu^3+^ emission.

The photostability of Ln^3+^-complex-doped di-ureasils was evaluated measuring the emission spectra every 15 min upon 295 and 343 nm irradiation during ~250 min. The results are shown in Figure S13a,b for the illustrative example of **d6Eu-1**. The irradiation time dependency of the ^5^D_0_→^7^F_2_ emission intensity is presented in Figure S13c (Supplementary Materials). After an irradiation time of ~250 min at 295 nm, the emission intensity drops by about 15%. However, by decreasing the irradiation energy (343 nm), the overall drop decreases to about 5%. For both irradiation wavelengths and during the study time interval, **d6Eu-1** displays a near linear photodegradation (−0.0575 and −0.0356%/min, for 295 and 343 nm, respectively), similar to what has been reported elsewhere [28].

## 4. Conclusions

In this study, organic–inorganic di-ureasil hybrids doped with Ln^3+^ complexes with the polycarboxylate ligand Oba and the ancillary ligand Phen were synthesized via an in-situ sol–gel technique via hydrolysis and condensation of the precursor in the presence of Ln^3+^ ions and the organic ligands. The resulting Ln^3+^-complex-doped d-U(600) hybrids were structurally and thermally characterized. The samples’ excitation and emission features reveal an efficient ligand-to-Ln^3+^ energy transfer, due to the absence of the ligands triplet-related emission. Indeed, these Ln^3+^-complex-based di-ureasils exhibit promising luminescent features, e.g., the Eu^3+^-based materials are bright red emitters displaying high quantum yields (up to 0.50 ± 0.05). The hybrid-related excited states play a crucial role in the population of the Eu^3^^+^ emitting levels through the Oba ligand, due to the high T_1_ energy level of Oba. Furthermore, the emission color can be fine-tuned either by the selection of adequate Ln^3+^ ions or by the variation of the excitation wavelength. White light emission with CIE coordinates of (0.33, 0.35) and a quantum yield of 0.03 ± 0.01 under 310 nm irradiation is observed in di-ureasils doped with Gd^3+^, Eu^3+^, and Tb^3+^ ions, since the Gd^3+^ incorporation enables the detection of the ligands/hybrid intrinsic emissions. In summary, the in-situ sol–gel method used in this work provides an elegant strategy to synthesize luminescent organic–inorganic hybrids containing efficient Ln^3+^ complexes with polycarboxylate ligands.

## Figures and Tables

**Table 1:** Emission quantum yield (*q*), ^5^D_0_ lifetime value (*τ*, ms), quantum efficiency (η), radiative (*k*_r_, ms^−1^) and non-radiative (*k*_nr_, ms^−1^) transition probabilities and number of water molecules (*n_w_*, ±0.1) of selected hybrids. The excitation wavelength (λ*_x_*, nm) is also indicated.

		*q*		τ	η	*k_r_*	*k_nr_*	*n_w_*
λ_x_ (nm)	275	295	325	295				
**dU6Eu-1**	35 ± 4	0.39 ± 0.04	26 ± 3	1.00 ± 0.05	0.48	0.487	0.518	0.2
**dU6Eu-2**	48 ± 5	0.50 ± 0.05	40 ± 4	0.92 ± 0.01	0.43	0.465	0.626	0.4
**dU6Eu-3**	12 ± 1			0.83 ± 0.01	0.37	0.441	0.762	0.5

## Supplementary Materials

The following are available online at http://www.mdpi.com/2073-4360/10/4/434/s1, Scheme S1. Molecular structures of (a) d-U(600), (b) Oba, and (c) Phen. Figure S1. XRD patterns of (1) d-U(600), (2) 2Eu(Oba)_1.5_(Phen)_1.5_@dU6, (3) 6Eu(Oba)_1.5_(Phen)_1.5_@dU6, and (4) 10Eu(Oba)_1.5_(Phen)_1.5_@dU6. Figure S2. FT-IR spectra of Eu(NO_3_)_3_, Oba, and Phen. Figure S3. (a) ^29^Si MAS and b) ^13^C CP MAS NMR spectra of 6Eu(Oba)_1.5_(Phen)_1.5_@dU6. Figure S4. TG curves for d-U(600) and 6Eu(Oba)_1.5_(Phen)_1.5_@dU6. Figure S5. Contour plots of (a) HOMO and (b) LUMO of Oba with an isovalue of 0.045. Figure S6. Individual atomic contributions to the electron density distributions in a) HOMO and b) LUMO of Oba. Figure S7. UV-visible absorption spectra of Oba in DMF solution. Figure S8. Phosphorescence emission spectrum of 6Gd(Oba)_1.5_@dU6 recorded under 270 nm excitation at 12 K. Figure S9. Emission spectra of (a) 6Eu(Oba)_1.5_@dU6, (b) 6Eu(Phen)_1.5_@dU6, (c) 6Eu(Oba)_1.5_(Phen)_1.5_@dU6, and (d) 6Tb(Oba)_1.5_(Phen)_1.5_@dU6. Figure S10. a) Excitation (λ_em_ = 617 nm) and b) emission (λ_ex_ = 285, 315, 330 and 393 nm) spectra of 6Eu@dU6 hybrid. Figure S11. Decay curves for (a) 6Eu@dU6, (b) 6Eu(Oba)_1.5_@dU6 (c) 6Eu(Phen)_1.5_@dU6, (d) 6Eu(Oba)_1.5_(Phen)_1.5_@dU6, and e) 6Tb(Oba)_1.5_(Phen)_1.5_@dU6. Figure S12. Emission spectra of d-U(600) hybrid depending on excitation wavelength. Figure S13. Photostabilities of d6Eu-1. (a,b) emission spectra under successive 295 and 343 nm irradiation, (c) time dependences of emission intensities under 295 and 343 nm irradiation. Table S1. Dopant components of prepared samples. Table S2. Computational FMOs, absorption wavelength, oscillator strengths, and S_1_ and T_1_ energy levels. Table S3. Calculated *R* values, quantum efficiency (*η*), and coordination water (*n_w_*). Table S4. All measured quantum yield values in this paper.
