# Supplementary material for: Highly Efficient Luminescent Polycarboxylate Lanthanide Complexes Incorporated into Di-Ureasils by an In-Situ Sol—Gel Process
Source: Polymers (Basel). 2018 Apr 13;10(4):434. doi: 10.3390/polym10040434 (PMC6415227; doi:10.3390/polym10040434)
Supplement: Supplementary file 1 [file polymers-10-00434-s001.pdf]

polymers

MDPI

Supplementary Materials

Highly Efficient Luminescent Polycarboxylate Lanthanide Complexes Incorporated into Di-Ureasils by an In-Situ Sol–Gel Process

Ming Fang, Lianshe Fu, Sandra F. H. Correia, Rute A. S. Ferreira, and Luís D. Carlos

Department of Physics, CICECO - Aveiro Institute of Materials, University of Aveiro, 3810-193 Aveiro, Portugal

1. Experimental Details

The powder X-ray diffraction (XRD) patterns were recorded in the 2θ range spanning from 3.5° to 60.0° by using a Ragaku-D/Max 2500 diffractometer system under exposure of Cu K_{α} radiation (1.54 Å) at room temperature.

^{29}Si magic-angle spinning (MAS) and ^{13}C cross-polarization (CP) MAS nuclear magnetic resonance (NMR) spectra were obtained by using a Bruker Avance III 400 spectrometer at 400 MHz. For recording ^{29}Si MAS signal, the measurement parameters were set at time between scans: 60.0 s, plus length: 2.1 μs, flip angle: 40.0° and 5 KHz spinning rate. For recording ^{13}C CP MAS signal, the measurement parameters were set at time between scans: 1.5 s, pulse length: 3.0 μs, CP contact time: 3500.0 μs and 12 KHz spinning rate.

Thermogravimetric (TG) measurements were performed from 75 to 800 ^{o}C with a 10 ^{o}C/min heating speed under air atmosphere on SDT 2960 analyzer (Shimadzu, Japan).

2. Experimental Results

2.1. Powder XRD Patterns

In Figure S1, it gives out the XRD patterns of d-U(600), 2Eu(Oba)$_{1.5}$(Phen)$_{1.5}$@dU6, 6Eu(Oba)$_{1.5}$(Phen)$_{1.5}$@dU6, and 10Eu(Oba)$_{1.5}$(Phen)$_{1.5}$@dU6 correspondingly related to curve 1, 2, 3 and 4, respectively. The coherent length L is calculated based on Scherrer equation [S1], $L=I\lambda/(A\cos\theta)$, in which I and A are intensity and integrated area of the peak around 21.0°, respectively, in radians unit, yielding L=14.7 ± 2.0 Å for 6Eu(Oba)$_{1.5}$(Phen)$_{1.5}$@dU6 and 14.6 ± 2.0 Å for dU(600), which is very close to that previously found for the di-ureasil host [S2]. The fact that there are no significant changes in the patterns after the *in-situ* formation of the complex, suggests that the local structure of the hybrid host remains essentially unaltered. We also note that, the diffraction pattern of the 6Eu(Oba)$_{1.5}$(Phen)$_{1.5}$@dU6 also reveals very low intense diffraction peaks at 18.0°, 20.0° and 24.0° which also present in the Oba diffraction pattern, suggesting the clustering of uncoordinated Oba in the gelation process, despite the fact that a transparent solution of Oba in DMF solution by ultrasonic treatment was obtained before added to d-UPTES(600) precursor. The presence of Oba-related diffraction peaks is also more evident as Eu^{3+} concentration increases from 2–10 mol%.

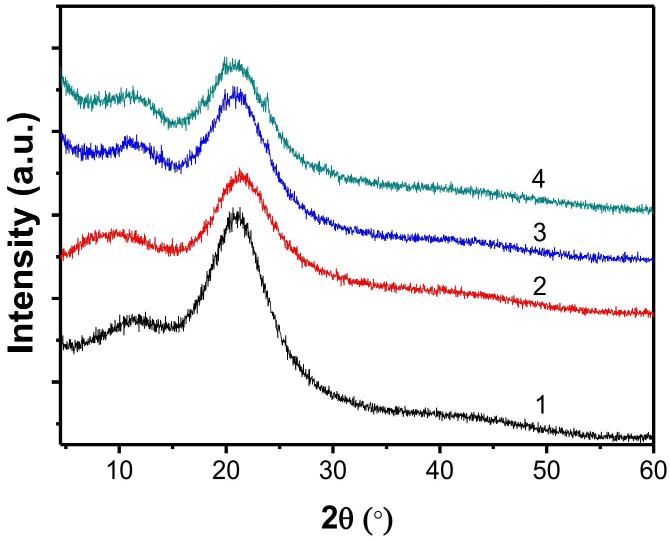

Figure S1. XRD patterns of 1) d-U(600), 2) 2Eu(Oba)$_{1.5}$(Phen)$_{1.5}$@dU6, 3) 6Eu(Oba)$_{1.5}$(Phen)$_{1.5}$@dU6, and 4) 10Eu(Oba)$_{1.5}$(Phen)$_{1.5}$@dU6.

2.2. FT-IR Spectroscopy

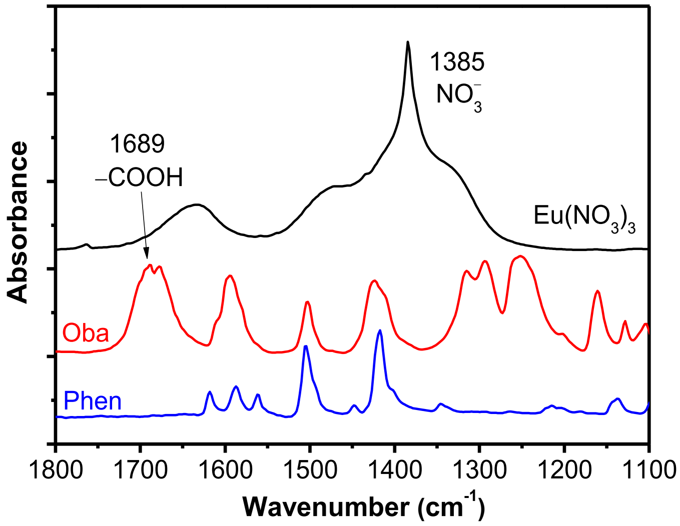

Figure S2. Infrared spectra of powders of Eu(NO$_3$)$_3$, Oba and Phen.

2.3. ²⁹Si MAS and ¹³C CP NMR Spectra

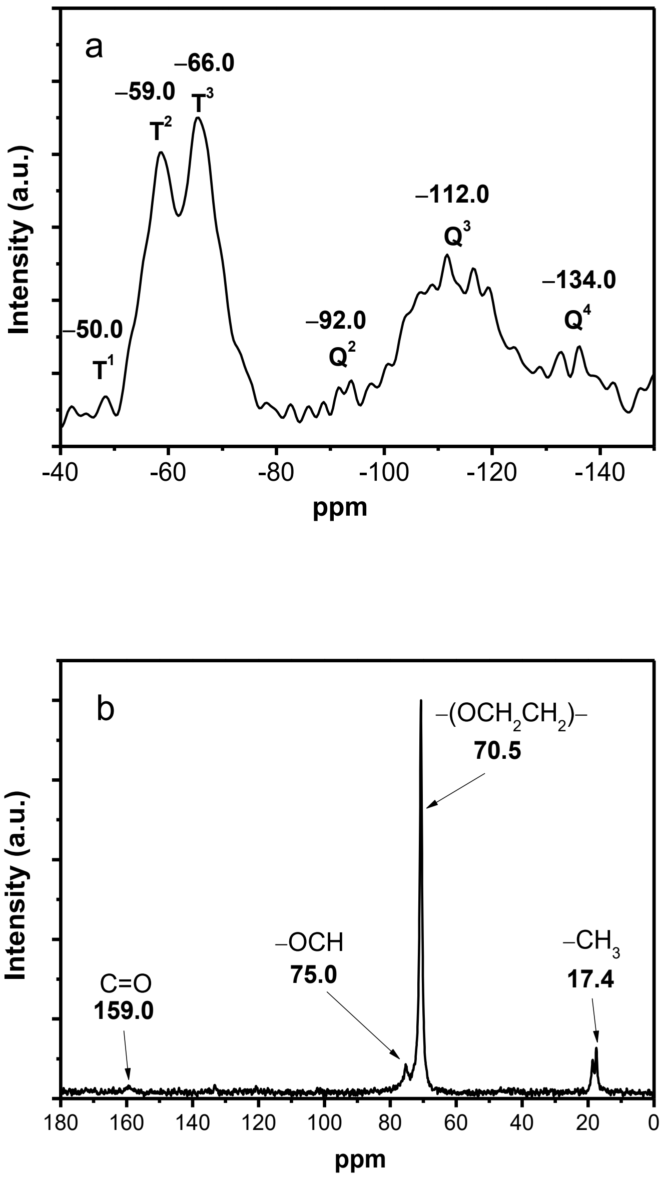

Figure S3. a) ^{29}Si MAS and b) ^{13}C CP MAS NMR spectra of 6Eu(Oba)$_{1.5}$(Phen)$_{1.5}$@dU6.

2.4. TG and Thermal Stability Analyses

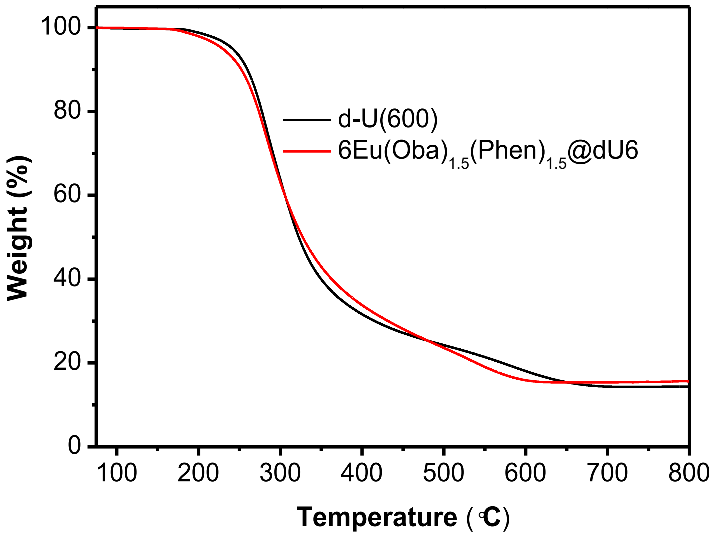

Figure S4. TG curves for d-U(600) and $6Eu(Oba)_{1.5}(Phen)_{1.5}$@dU6.

2.5. DFT/TD-DFT Calculations

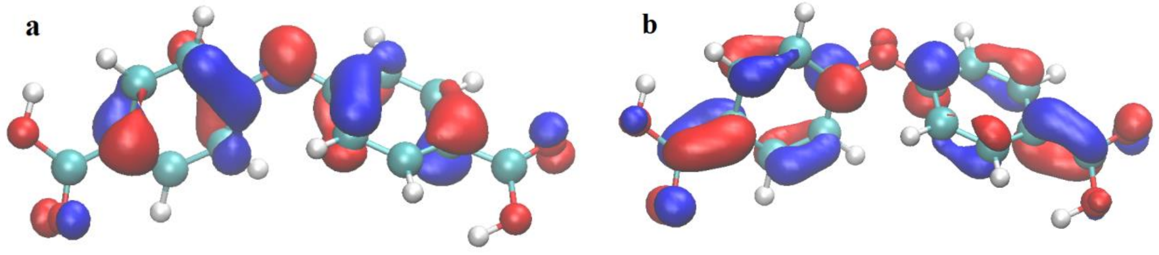

Figure S5. Contour plots of a) HOMO and b) LUMO of Oba with isovalue of 0.045.

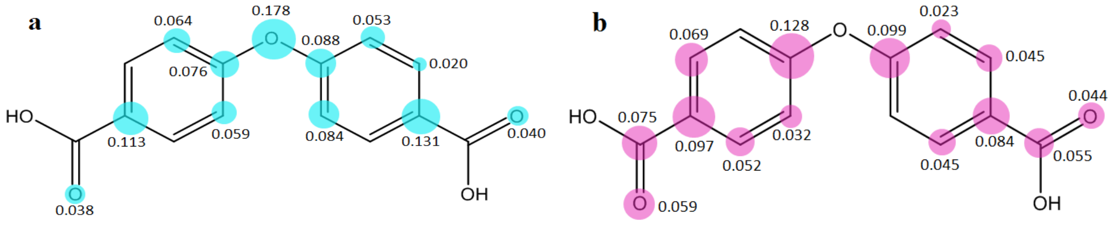

Figure S6. Individual atomic contributions to the electron density distributions in a) HOMO and b) LUMO of Oba, the areas of the circles are proportional to the atomic contributions, and only contributions greater than 0.020 are shown.

2.6. UV/vis Absorption Spectroscopy

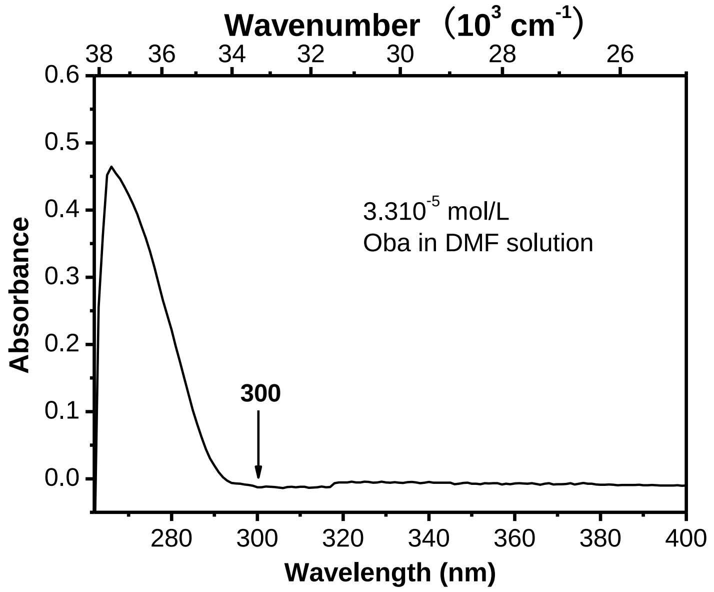

Figure S7. UV–Vis absorption spectrum of Oba in DMF solution (3.3×10^{-5} mol/L).

2.7. Photoluminescence

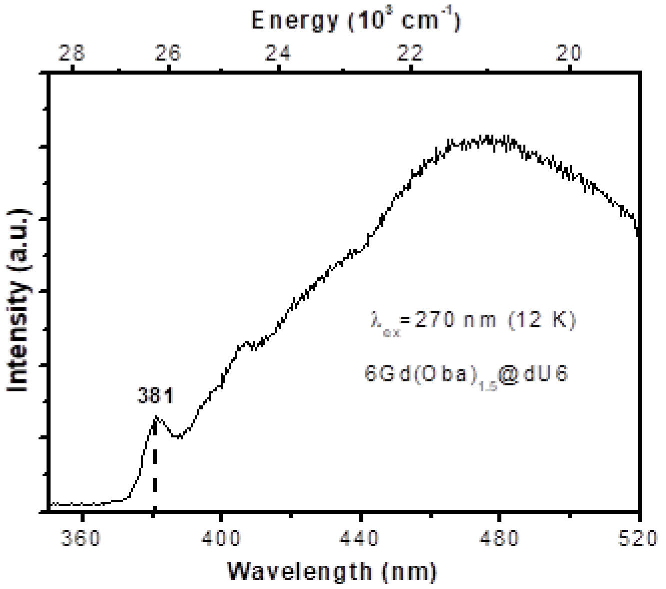

Figure S8. Phosphorescence emission spectrum of 6Gd(Oba)$_{1.5}$@dU6 recorded under 270 nm excitation at 12 K.

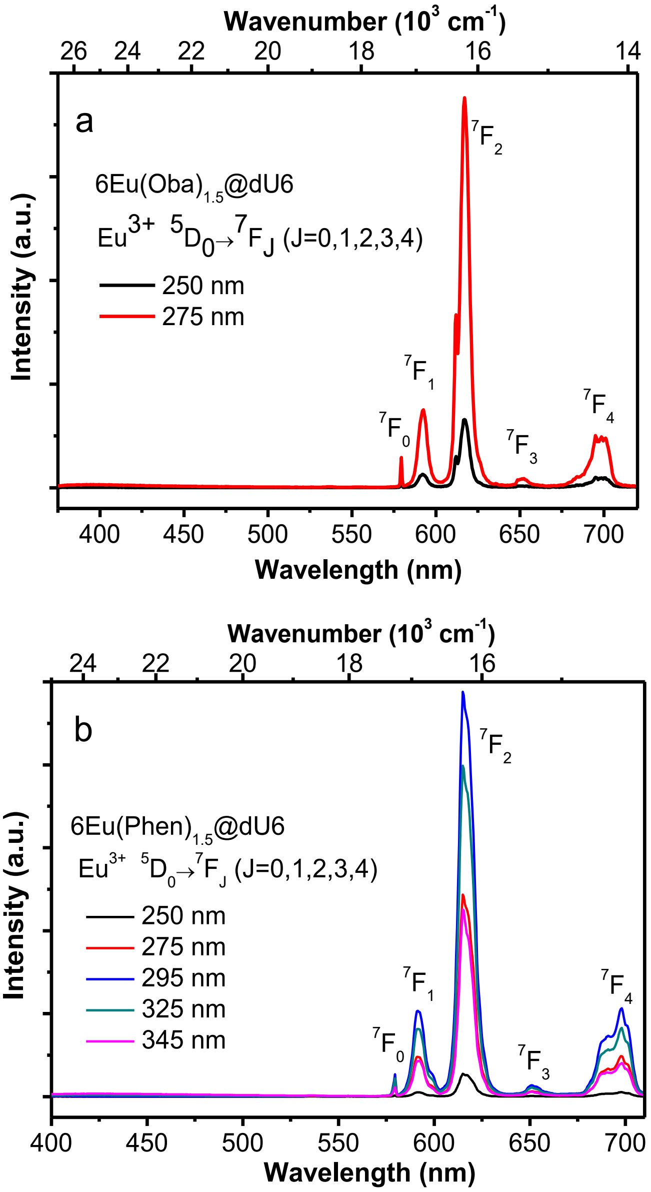
Wavenumber (10^3 cm^-1)
a
6Eu(Oba)1.5@dU6
Eu3+ 5D0→7FJ (J=0,1,2,3,4)
250 nm
275 nm
7F0
7F1
7F2
7F3
7F4
Intensity (a.u.)
Wavelength (nm)
b
6Eu(Phen)1.5@dU6
Eu3+ 5D0→7FJ (J=0,1,2,3,4)
250 nm
275 nm
295 nm
325 nm
345 nm
7F0
7F1
7F2
7F3
7F4
Intensity (a.u.)
Wavelength (nm)

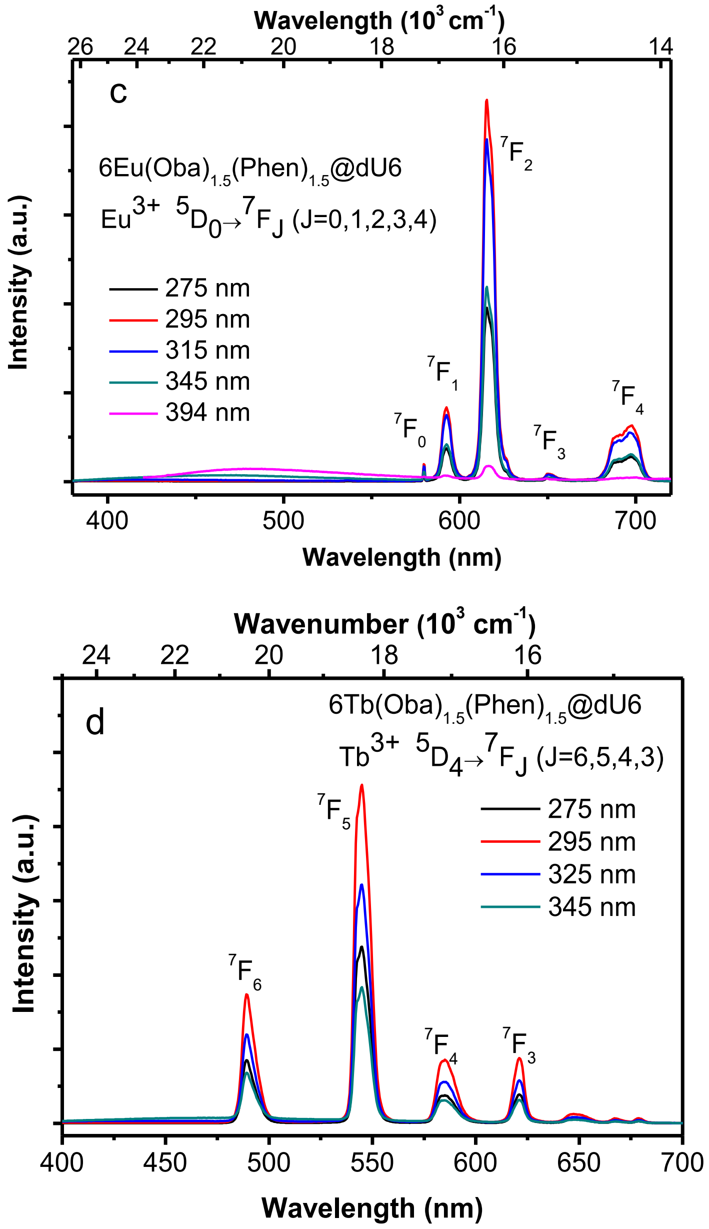

Figure S9. Emission spectra of a) 6Eu(Oba)$_{1.5}$@dU6, b) 6Eu(Phen)$_{1.5}$@dU6, c) 6Eu(Oba)$_{1.5}$(Phen)$_{1.5}$@dU6 and d) 6Tb(Oba)$_{1.5}$(Phen)$_{1.5}$@dU6

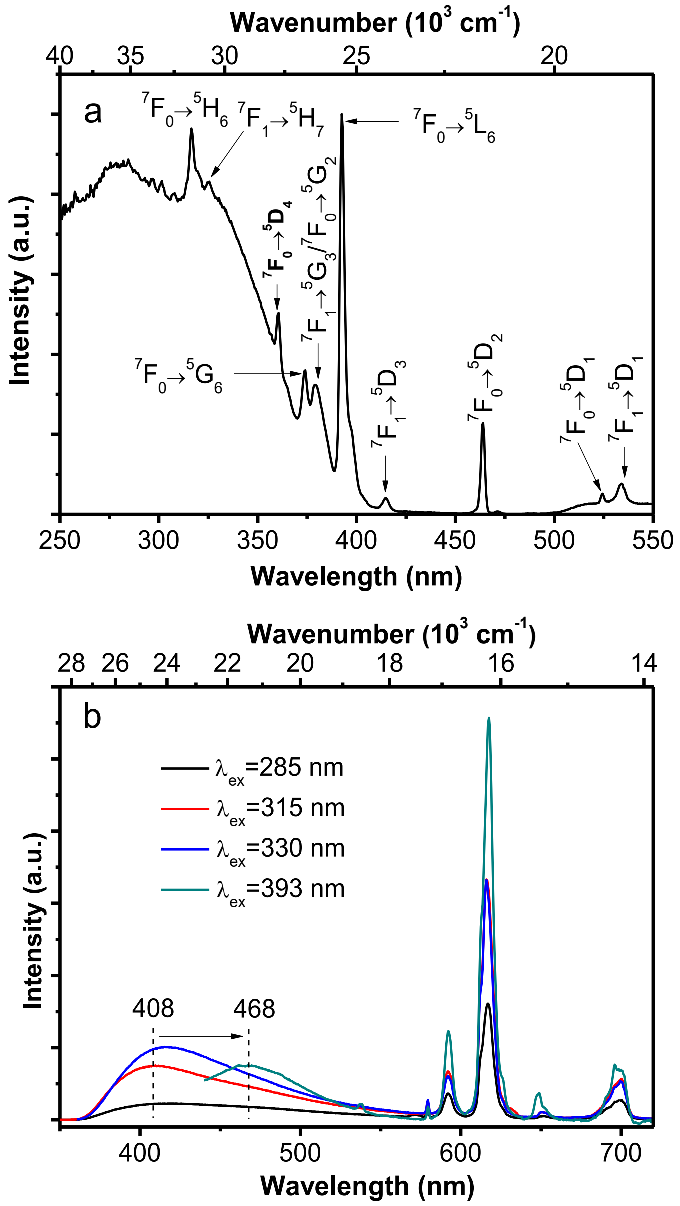

Figure S10. a) Excitation (λ_{em}=617 nm) and b) emission (λ_{ex}=285, 315, 330 and 393 nm) spectra of 6Eu@dU6 hybrid.

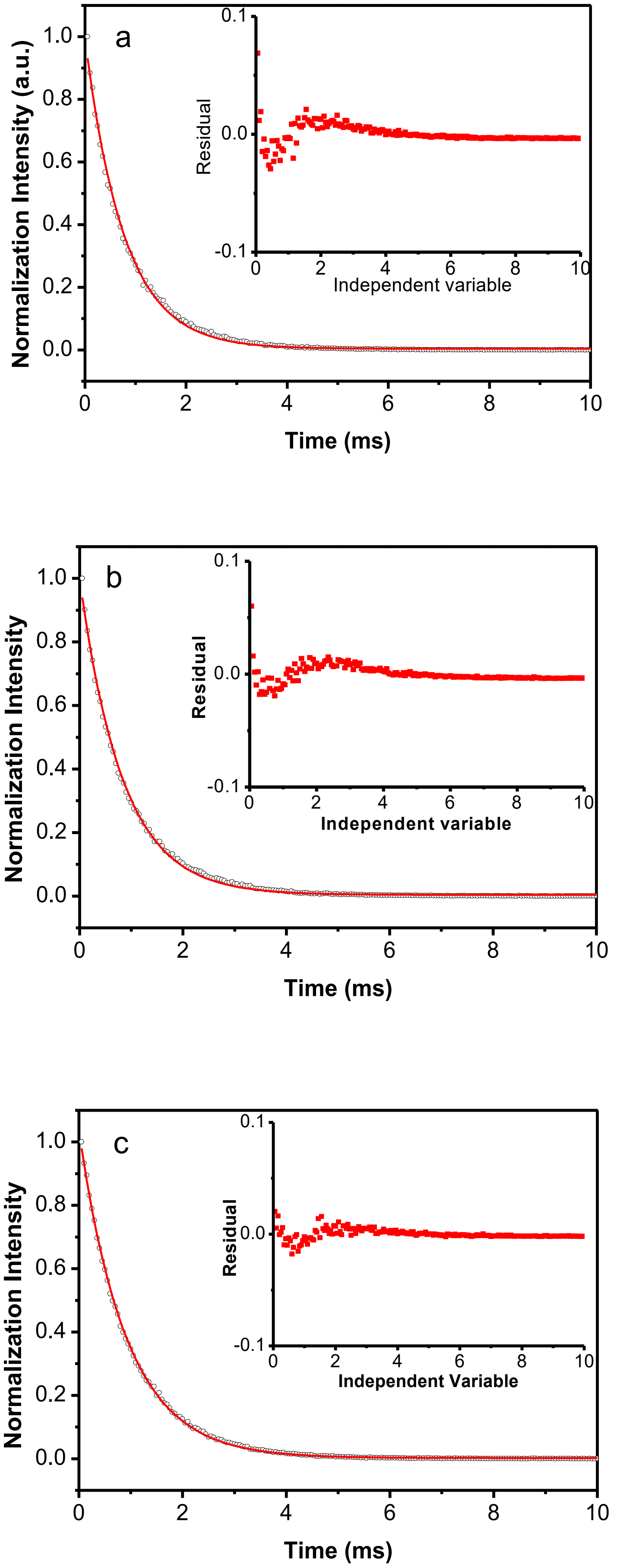
a
Normalization Intensity (a.u.)
Time (ms)
Residual
Independent variable
b
Normalization Intensity
Time (ms)
Residual
Independent variable
c
Normalization Intensity
Time (ms)
Residual
Independent Variable

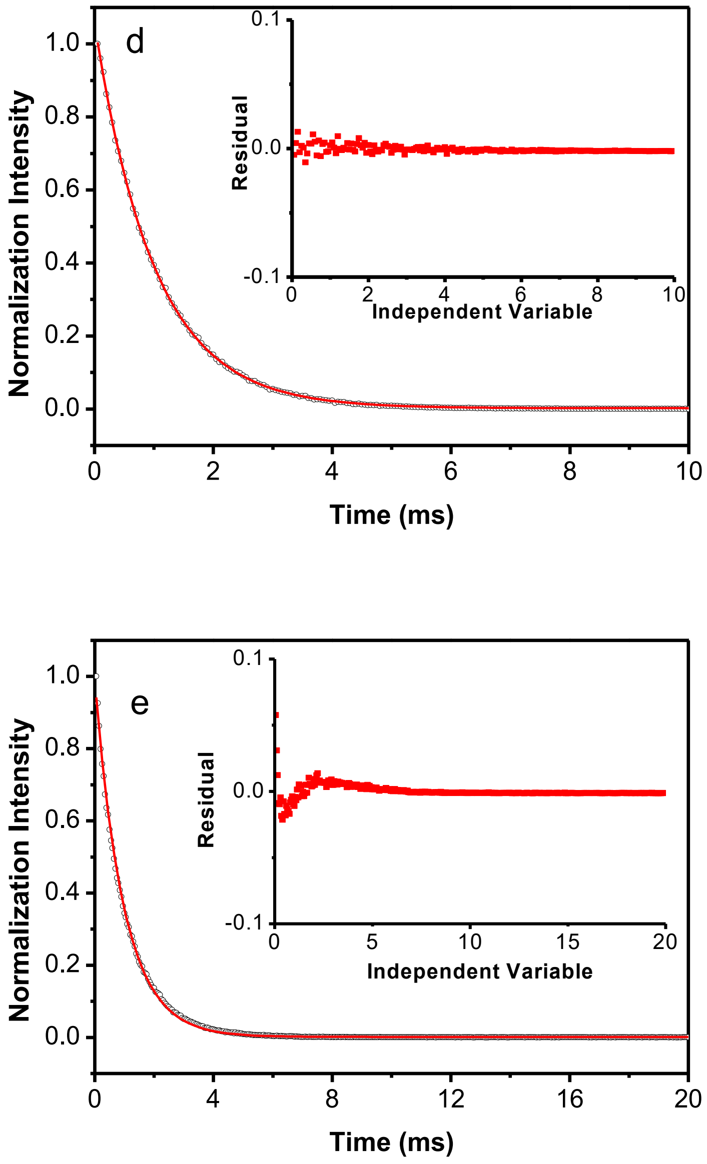

Figure S11. Decay curves for a) 6Eu@dU6, b) 6Eu(Oba)$_{1.5}$@dU6, c) 6Eu(Phen)$_{1.5}$@dU6, d) 6Eu(Oba)$_{1.5}$(Phen)$_{1.5}$@dU6 and e) 6Tb(Oba)$_{1.5}$(Phen)$_{1.5}$@dU6. The lines are fits to the experimental data using mono-exponential functions (r^2>0.9974) yielding τ=0.772, 0.831, 0.917, 1.005 and 0.970 s.

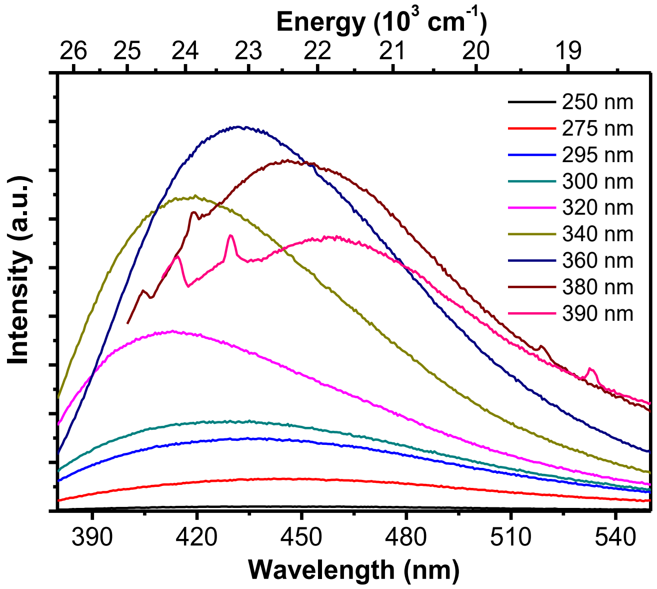

Figure S12. Excitation wavelength dependence of the d-U(600) emission.

2.7. Photostability

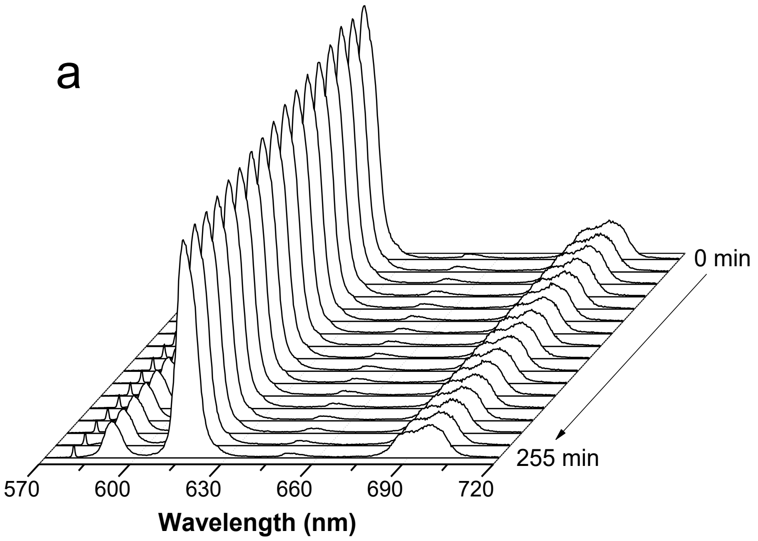

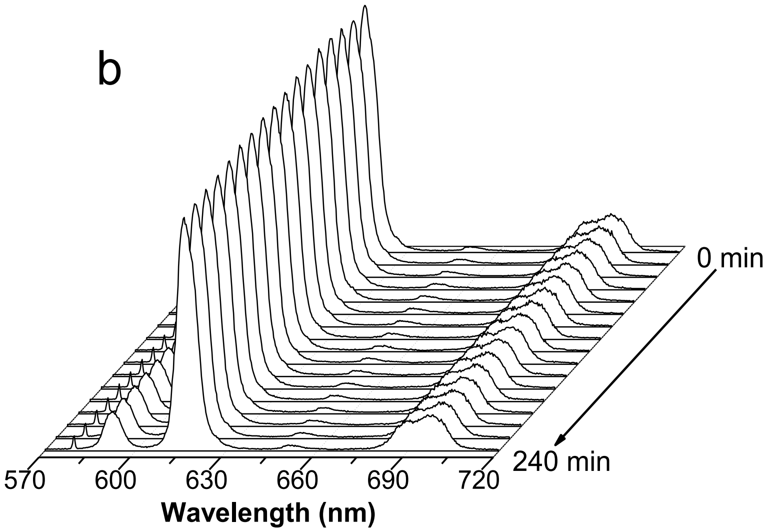

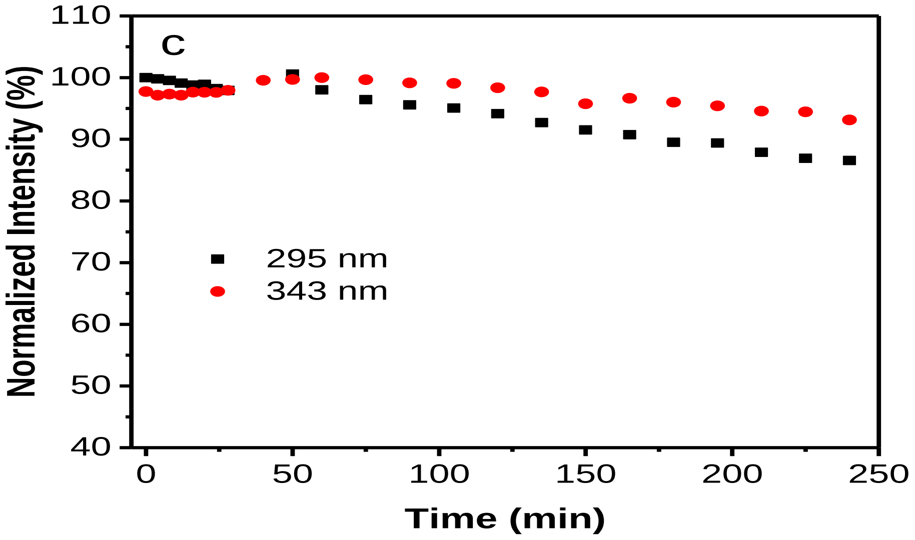

Figure S13. Photostability of **d6Eu-1**. Emission spectra under continuous irradiation at a) 295 nm and b) 343 nm. c) Time dependence of the maximum emission intensity of the $^5D_0 \rightarrow {}^7F_2$ transition under continuous irradiation at 295 and 343 nm.

Calculation of the radiative (k_r) and non-radiative probability constants (k_{nr}), quantum efficiency (η) as well as the number of water molecules (n_w) coordinated to the Eu^{3+} ions.

The radiative probability constant, k_r, can be calculated from the relative intensities of the $^5D_0 \rightarrow {}^7F_J$ (J = 0–4) transitions and it can be expressed as [S3,S4]:

$$k_r = (A_{0-1} E_{0-1} / S_{0-1}) \sum_{j=0}^{4} (S_{0-J} / E_{0-J}) \quad (1)$$

where A_{0-1} is Einstein's coefficient of spontaneous emission between $^5D_0$ and $^7F_1$ levels, and E_{0-J} and S_{0-J} are the energy and the integrated intensity of the $^5D_0 \rightarrow {}^7F_J$ transitions, respectively. Since the $^5D_0 \rightarrow {}^7F_1$ transition does not depend on the local ligand field, it can be used as a reference for the

whole spectrum. Since *in vacuo*, $(A_{0-1})_{vac}$=14.65 s^{-1}, if an average index of refraction n equal to 1.5 was considered, the value of $A_{0-1}=n^3A_{(0-1)vac} \approx 50\ s^{-1}$ [S5–S7].

The non-radiative probability constant, k_{nr}, can be obtained from the experimental $^5D_0$ lifetime:

$$k_{nr}= \tau_{exp}{}^{-1}- k_r \tag{2}$$

and the quantum efficiency, η, is obtained from the following formula.

$$\eta = k_r/(k_r+k_{nr}) \tag{3}$$

According to Horrocks [S8], n_w can be estimated from the experimental decay time by the empirical formula:

$$n_w=1.1\times(k_{exp}-k_r-0.31) \tag{4}$$

Scheme 1

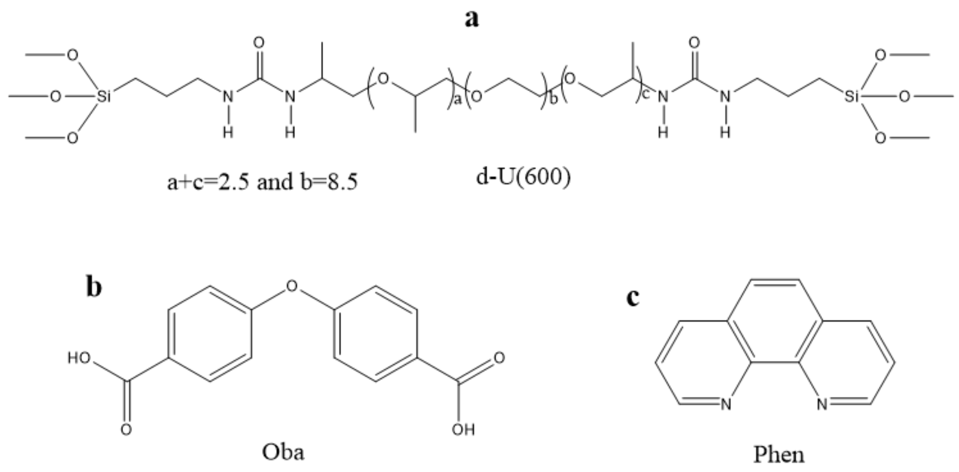

Scheme S1. Molecular structures of a) d-U(600), b) Oba and c) Phen.

Table S1. Dopant components of the prepared samples.

Samples	$Eu(NO_3)_3{\cdot}6H_2O$ (mg)	$Tb(NO_3)_3{\cdot}5H_2O$ (mg)	$Gd(NO_3)_3{\cdot}6H_2O$ (mg)	Oba (mg)	Phen (mg)	DMF (µL)
dU6						
6Oba@dU6				21.2		750
Phen@dU6					14.8	
6Eu@dU6	24.5					
$6Eu(Oba)_{1.5}$@dU6	24.5			21.2		750
$6Eu(Phen)_{1.5}$@dU6	24.5				14.8	
$2Eu(Oba)_{1.5}(Phen)_{1.5}$@dU6	8.2			7.1	5.0	500
$6Eu(Oba)_{1.5}(Phen)_{1.5}$@dU6	24.5			21.2	14.8	750
$10Eu(Oba)_{1.5}(Phen)_{1.5}$@dU6	40.8			35.4	24.7	1000
$6Tb(Oba)_{1.5}(Phen)_{1.5}$@dU6		23.9		21.2	14.8	750
$6Gd(Oba)_{1.5}$@dU6			20.4	21.2		750
$Gd_{0.91}Eu_{0.05}Tb_{0.04}(Oba)_{1.5}(Phen)_{1.5}$@dU6	0.6	0.5	9.3	21.2	14.8	500

For syntheses of all samples, 1.0 g of d-UPTES(600) precursor, 960.7 µL of EtOH and 98.7 µL of HCl (pH=2) were added.

Table S2. Computational FMOs, absorption wavelength, oscillator strengths, and S_1 and T_1 energy levels.

HOMO (eV)	LUMO (eV)	Gap (eV)	Absorption (nm)	*f*	Assignment	S_1 (nm/cm^{-1})	T_1 (nm/cm^{-1})
−7.01	−1.98	−5.03	276	0.404	HOMO→LUMO (98.5%)	313/31950	361/27700

Table S3. Calculated R values, quantum efficiencies and number of coordinated water molecules for selected di-ureasils.

Hybrids	6Eu(Oba)$_{1.5}$@dU6	6Eu(Phen)$_{1.5}$@dU6	6Eu(Oba)$_{1.5}$(Phen)$_{1.5}$@dU6
R	5.77	5.55	6.15
η	0.37	0.43	0.48
n_w	0.50	0.35	0.23
λ_{ex} (nm)	275	295	295

Table S4. Quantum yield values for selected di-ureasils.

Sample	λex (nm)	*q*
**dU6Eu-1**	325	0.26±0.03
	295	0.39±0.04
	275	0.350±0.04
**dU6Eu-2**	325	0.40±0.04
	295	0.50±0.05
	275	0.48±0.05
**dU6Eu-3**	275	0.12±0.01
**dU6Tb-1**	275	0.23±0.02
	285	0.21±0.02
	295	0.21±0.02
	305	0.16±0.02
	325	0.10±0.01
**dU6GdTbEu-1**	310	0.03±0.01

References

1. Guinier, A. X-ray diffraction in crystals, imperfect crystals and amorphous boldies; Dover: New York, **1994**; pp. 121–125.
2. Fu, L.S.; Ferreira, R.A.S.; Fernandes, M.; Nunes, S.; de Zea Bermudez, V.; Hungerford, G.; Rocha, J.; Carlos, L.D. Photoluminescence and quantum yields of organic/inorganic hybrids prepared through formic acid solvolysis. *Opt. Mater.* **2008**, *30*, 1058–1064.
3. Carlos, L.D.; Messaddeq, Y.; Brito, H.F.; Ferreira, R.A.S.; de Zea Bermudez, V.; Ribeiro, S.J.L. Full-color phosphors from europium(III)-based organosilicates. *Adv. Mater.* **2000**, *12*, 594–598
4. Ferreira, R.A.S.; Carlos, L.D.; Gonçalves, R.R.; Ribeiro, S.J.L.; de Zea Bermudez, V. Energy-transfer mechanisms and emission quantum yields in Eu^{3+}-based siloxane-poly(oxyethylene) nanohybrids. *Chem. Mater.* **2001**, *13*, 2991–2998.
5. Werts, M.H.V.; Jukes R.T.F.; Verhoeven, J.W. The emission spectrum and the radiative lifetime of Eu^{3+} in luminescent lanthanide complexes. *Phys. Chem. Chem. Phys.* **2002**, *4*, 1542–1548.
6. Hazenkamp, M.F.; Blasse, G. Rare-earth ions adsorbed onto porous-glass-luminescence as a characterizing tool. *Chem. Mater.* **1990**, *2*, 105–110.
7. Ribeiro, S.J.L.; Dahmouche, K.; Ribeiro, C.A.; Santilli, C.V.; Pulcinelli, S.H.J. Study of hybrid silica-polyethyleneglycol xerogels by Eu^{3+} luminescence spectroscopy. *J. Sol-Gel Sci. Technol.* **1998**, *13*, 427–432.
8. Supkowski, R.M.; Horrocks, W.D. On the determination of the number of water molecules, q, coordinated to europium(III) ions in solution from luminescence decay lifetimes. *Inorg. Chim. Acta* **2002**, *340*, 44–48.

© 2018 by the authors. Submitted for possible open access publication under the terms and conditions of the Creative Commons Attribution (CC BY) license (http://creativecommons.org/licenses/by/4.0/).
